# Supplementary material for: Media framing of childhood obesity: a content analysis of UK newspapers from 1996 to 2014
Source: BMJ Open. 2019 Apr 4;9(4):e025646. doi: 10.1136/bmjopen-2018-025646 (PMC6500306; doi:10.1136/bmjopen-2018-025646)

Supplementary figure 1 – Caption <Frequency of newspaper articles about generic, childhood, and adult obesity >

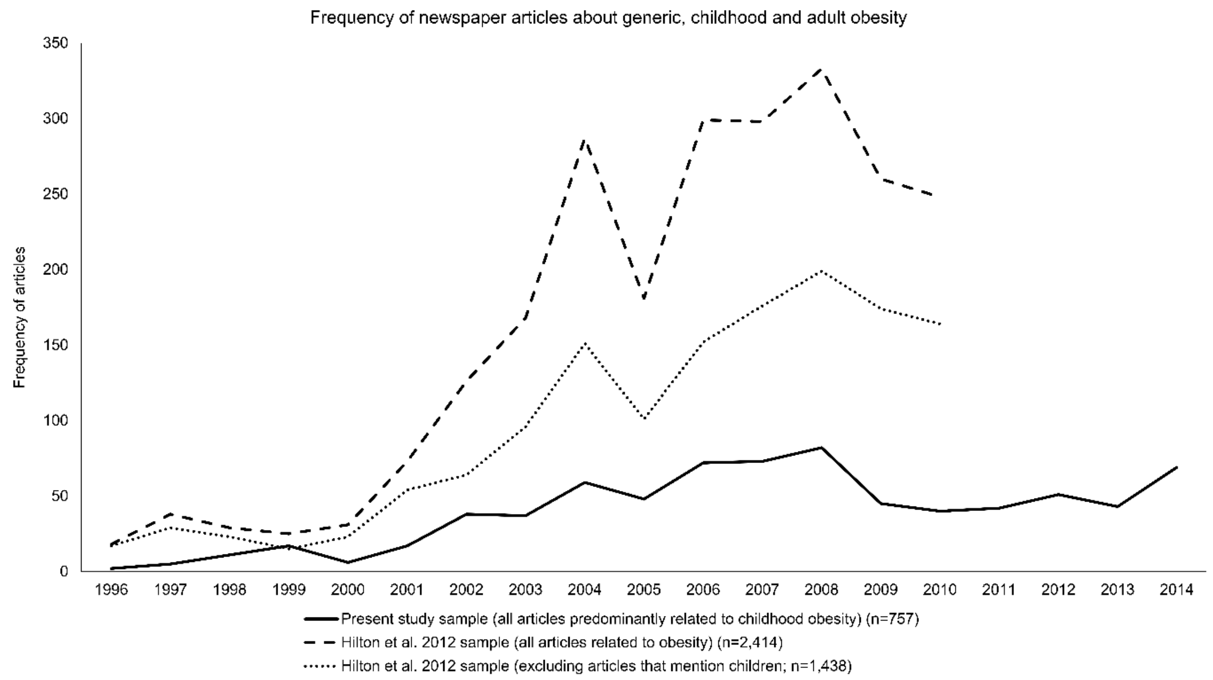

Supplementary figure 2 – Caption < Proportion of articles within years mentioning different categories of drivers and solutions about generic, childhoood, and adult obesity>

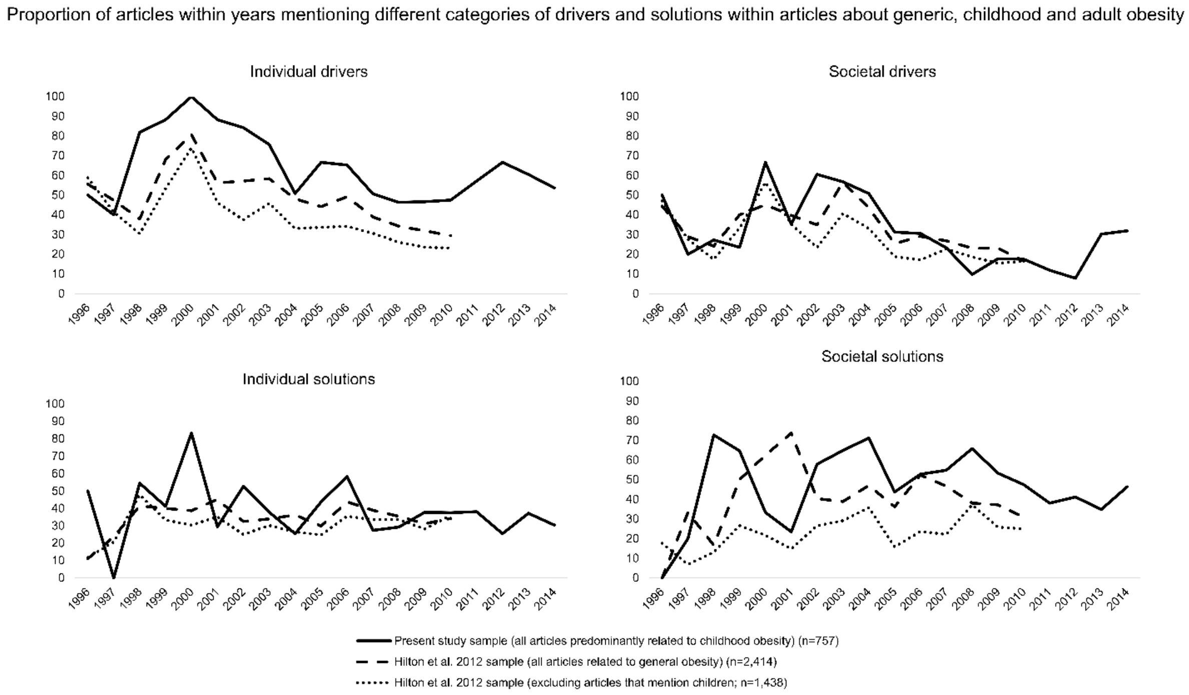

Supplement: Supplementary data [file bmjopen-2018-025646supp001.pdf]
