# Supplementary material for: Media framing of childhood obesity: a content analysis of UK newspapers from 1996 to 2014
Source: BMJ Open. 2019 Apr 4;9(4):e025646. doi: 10.1136/bmjopen-2018-025646 (PMC6500306; doi:10.1136/bmjopen-2018-025646)
Supplement: Author's manuscript [file bmjopen-2018-025646.draft_revisions.pdf]

BMJ Open is committed to open peer review. As part of this commitment we make the peer review history of every article we publish publicly available.

When an article is published we post the peer reviewers' comments and the authors' responses online. We also post the versions of the paper that were used during peer review. These are the versions that the peer review comments apply to.

The versions of the paper that follow are the versions that were submitted during the peer review process. They are not the versions of record or the final published versions. They should not be cited or distributed as the published version of this manuscript.

BMJ Open is an open access journal and the full, final, typeset and author-corrected version of record of the manuscript is available on our site with no access controls, subscription charges or pay-per-view fees (http://bmjopen.bmj.com).

If you have any questions on BMJ Open's open peer review process please email info.bmjopen@bmj.com

BMJ Open

Media framing of childhood obesity: a content analysis of UK newspapers from 1996-2014

Journal:	*BMJ Open*
Manuscript ID	bmjopen-2018-025646
Article Type:	Research
Date Submitted by the Author:	27-Jul-2018
Complete List of Authors:	Nimegeer, Amy; Glasgow University, MRC/CSO Social and Public Health Sciences<br>Patterson, Chris; University of Glasgow, MRC/CSO Social & Public Health Sciences Unit<br>Hilton, Shona; University of Glasgow, MRC/CSO Social & Public Health Sciences Unit
Keywords:	Health policy < HEALTH SERVICES ADMINISTRATION & MANAGEMENT, MEDICAL JOURNALISM, Community child health < PAEDIATRICS, PAEDIATRICS, PUBLIC HEALTH, STATISTICS & RESEARCH METHODS

SCHOLARONE™
Manuscripts

For peer review only

TITLE: Media framing of childhood obesity: a content analysis of UK newspapers from 1996-2014

AUTHORS: Amy Nimegeer[1], Chris Patterson[1], Shona Hilton[1]

AFFILIATION: [1]MRC/CSO Social and Public Health Sciences Unit, University of Glasgow, 200 Renfield Street, Glasgow, G2 3QB, United Kingdom

KEYWORDS: Obesity, Children, Media, content analysis

RUNNING TITLE: UK Media framing of childhood obesity

CONTACT INFORMATION:

Dr Amy Nimegeer, MRC/CSO Social and Public Health Sciences Unit, University of Glasgow, 200 Renfield Street, Glasgow, G2 3QB, United Kingdom

amy.nimegeer@glasgow.ac.uk

Emails for additional authors: chris.patterson@glasgow.ac.uk ; shona.hilton@glasgow.ac.uk

WORD COUNT (excluding cover page, abstract, references, tables, and figures): 4166

For peer review only

Abstract

Objective and Setting: Media can influence public and policymakers' perceptions of causes and solutions of public health issues through selective presentation and framing. To understand the salience and framing of childhood obesity across 19 years of UK national newspaper content.

Design and Outcome Measures: Quantitative content analysis of 757 articles about childhood obesity obtained from six daily and five Sunday newspapers. Articles were coded manually for definitions, drivers and potential solutions. Data were analysed statistically, including analysis of time trends and variations.

Results: The frequency of articles grew from a low of two in 1996 to a peak of 82 in 2008, then declined to 40 in 2010. Alarmist headlines (21.8%) greatly outnumbered reassuring headlines (2.9%). Individual-level drivers (59.8%) and solutions (36.5%) were mentioned more frequently than societal-level drivers (28.3%) and solutions (28.3%) across the sample, but societal solutions were mentioned more frequently during the final eight years, coinciding with a marked decline in overall frequency of articles.

Conclusions: Increased focus on societal solutions aligns with public health goals, but coincides with a reduction in media salience. Those advocating public policy solutions to childhood obesity may benefit from seeking to raise the issue's media profile while continuing to promote structural ways of conceptualising obesity.

For peer review only - http://bmjopen.bmj.com/site/about/guidelines.xhtml

Article Summary

Strengths and Limitations of this Study

Strengths

- Methodology includes systematic analysis of a large sample of nineteen years of UK national newspaper coverage, facilitating statistical understandings of media frames of childhood obesity, including definitions, drivers and solutions
- Features robust manual coding and links to pre-existing dataset to strengthen analysis

Weaknesses

- Quantitative media content analysis is inherently less nuanced than qualitative analysis and our analysis excluded images which may influence readers' interpretations of the media texts
- Content analysis makes clear which messages are produced by media but cannot tell us how these messages are received by audiences

Funding Statement

AN, CP and SH's time for this research was funded by the Informing Healthy Public Policy programme (MC_UU_12017-15 and SPHSU15) of the MRC/CSO Social and Public Health Sciences Unit, University of Glasgow. The funding bodies had no role in the design, collection, analysis or interpretation of this study.

Competing Interests

SH, CP and AN declare no conflicts of interest. All three authors work at the MRC/CSO Social and Public Health Sciences Unit, University of Glasgow, which receives joint funding from the Medical

For peer review only - http://bmjopen.bmj.com/site/about/guidelines.xhtml

Research Council and Chief Scientist Office. Their time on this research was paid for by that core funding.

Acknowledgements

The authors would like to thank Dr Jonathan Olsen of the MRC/CSO Social and Public Health Sciences Unit, University of Glasgow, for his advice regarding the conduct of multiple logistic regression analysis. We would also like to acknowledge the contribution of Dr Marie Löf and Dr Mark Daku to the design of this study.

For peer review only

For peer review only - http://bmjopen.bmj.com/site/about/guidelines.xhtml

Introduction

Childhood obesity has been described as an international epidemic due to its high prevalence and rapid growth in numerous countries(1). Globally, 13.4% of girls and 12.9% of boys in Low Income Countries, and 22.6% of girls, and 23.8% of boys in High Income Countries (classified by the World Bank) were overweight or obese in 2013(2). In England, one fifth of children in Reception year (age 4-5), and one third in Year 6 (age 10-11), were overweight or obese in 2015/2016 (3).In Scotland, 28% of children aged 2-15 were classified as 'at risk of' overweight or obesity in 2015 (4).Childhood obesity has a broad range of short- and long-term health consequences (1), tends to predict adolescent and adult obesity (5), and is socioeconomically patterned(3). For these reasons childhood obesity has been identified as a health priority for the UK and its devolved governments(6, 7).

Childhood obesity is a complex problem, with a complex set of drivers and potential solutions ranging from the individual to the environmental(8). Ebbeling and colleagues (1) identify a wide range of causes, but argue that the problem *"can be primarily attributed to adverse environmental factors"*, and identify a need for *"straightforward, if politically difficult"* solutions spanning homes, schools, the built environment, health care, marketing, media and politics. This multi-level package of solutions echoes Friedman's assertion that a 'full-court press' targeting 'every dimension of the problem' is necessary(9). However, while academia and public health are united on the need to target the obesogenic environment, Swinburn and colleagues (10) state that *"governments have largely abdicated the responsibility for addressing obesity to individuals, the private sector, and non-governmental organisations"*(10), potentially due to anticipated or actual resistance, not just from corporations, but also electorates (9, 11).Indeed, public opinion research conducted in the US (12)and Germany (13) suggests that, while publics are in favour of tackling childhood obesity, they demonstrate less enthusiasm for regulative environmental interventions such as taxation. Hilbert characterises the German population as *"ready for obesity prevention"*, but in need of education about the definition, prevalence and causes of obesity (12).

For peer review only - http://bmjopen.bmj.com/site/about/guidelines.xhtml

The media represent a key influence on public perceptions of health issues and policies, setting the public agenda by granting different levels of prominence to different topics(14), and influencing how those issues are understood by building frames (focuses of attention) that include constructions of problems, affected groups, drivers and solutions (15, 16). The influence of framing is well established in relation to obesity. Researchers have used experimental designs to demonstrate that 'individualised' representations of childhood obesity tend to lead participants to assign greater blame to individuals and exhibit less support for environmental regulation(17), and that different representations of the consequences of childhood obesity can influence participants' attitudes towards policies (18). Similarly, Barry and colleagues demonstrated that people's perceptions of obesity (as communicated through agreement with metaphor-based descriptions of obesity) predict their support for public policy interventions, illustrating how, for example, framing obesity as being driven by industry manipulation may lead to increased support for a 'junk-food tax'(19).

The media are frequently accused of contributing to obesity, particularly childhood obesity, through its associations with sedentary behaviour, advertising of unhealthy commodities, promotion of unrealistic body image, and other mechanisms(20). Many researchers have studied media representations of obesity in general (21, 22, 23, 24, 25, 26, 27, 28, 29, 30, 31, 32, 33), but relatively few have focused specifically on representations of childhood obesity, and these have been primarily in the US and Australia. Barry and colleagues (34) studied US print and television news framing of childhood obesity, observing that coverage of the issue grew between 2000 and 2009, and that individual-level behavioural solutions to obesity were dominant, particularly on television. Similarly, Hawkins and Linvill (35) studied US newspaper framing of childhood obesity over three discrete time periods in 1991, 2001 and 2006, and identified a predominant focus on individual-level factors (both individual children and their parents) in representations of both causes and solutions. Kalin and Fung (36) analysed Spanish-language US parenting magazines' representations of childhood obesity prevention and control, identifying greater focus on parental behaviour-change than system-level solutions, with a low level of recognition of social contextual factors, and recommend that health

For peer review only - http://bmjopen.bmj.com/site/about/guidelines.xhtml

professionals engage with the media to reframe coverage of the problem in environmental, rather than behavioural terms.

Bastian(37) analysed representations of childhood obesity in both Australian newspapers and academic literature in 2009, identifying predominantly individual framing within the media, compared to a social-structural framing in academic literature. Bastian (37) recommends that public health professionals work to redirect media attention towards structural drivers of childhood obesity. Maher and colleagues (38)analysed constructions of maternal responsibilities within Australian media coverage of childhood obesity, concluding that the dominant framing *"individualises maternal and child relationships rather than seeing mothering as embedded in broader social and economic structures"*, serving a neo-liberal agenda by diminishing the responsibility of wider society. This is consistent with the disproportionate focus on individual-level solutions identified by others (34, 35, 36, 37). While coverage of obesity in both adults and children appears to be characterised by individual-framing, it is notable that with adult obesity that individual responsibility is assigned to the person with obesity, while in childhood obesity that responsibility is predominantly assigned to parents, particularly mothers(39, 40). This distinction may complicate direct comparison between adult and child obesity, and the culturally-ingrained nature of the concept that parents (or mothers) are solely responsible for their children's healthcare may represent a discursive obstacle to attempts to assign environmental solutions to childhood obesity.

The aim of this study is to further understandings of media representations of childhood obesity in the UK context, using an approach informed by media framing theory(15, 16), analysing definitions of the problem and constructions of drivers and solutions. This is important because, while childhood obesity in the UK shares many similarities with that of other countries, the UK context differs in terms of several elements including health service structure and media environment. The analysis will have dual foci: the evolution of coverage between 1996 and 2014, and the relative salience of individual and societal constructions of the drivers of, and potential solutions to,

For peer review only - http://bmjopen.bmj.com/site/about/guidelines.xhtml

childhood obesity. To our knowledge, this research will be the first empirical analysis of UK media framing of childhood obesity specifically. This paper comprises the UK portion of a multi-country research project, the other parts of which will be reported in separate papers.

Methods

The media content analysis methods used were predominantly based on Hilton and colleagues' prior study(21) of UK newspaper framing of obesity in the general population, adapted for this study's focus on childhood obesity. This paper reports UK data that was part of a wider study that examined childhood obesity media coverage in two other international contexts; Sweden and the United States.

Sampling

A set of six daily newspapers and five Sunday newspapers with high circulation figures(41) were chosen. Table 1 lists these publications and indicates their political alignments and the markets (or 'genres') that they occupy. Markets were defined as tabloid (typically sensationalist and politically diverse, with predominantly working-class readerships), middle-market tabloid (centre-right content with predominantly older, middle-class readerships) and quality (serious tone with predominantly middle-class readerships), using a typology used in prior studies of UK newspaper content(21).A sample period of 1996 to 2014 was chosen to encompass the time period covered in prior research (21), in addition to a further four years of coverage that was extended to align with the time period covered by the other countries in our wider study (which will be described fully in a separate publication).

Identifying relevant articles from the chosen publications involved an initial database search, followed by manual filtering of search results. The Nexis database was searched for the presence of both the term *'obesity' OR 'obese' OR 'fat'* and the term *'child' OR 'children' OR 'kid' OR kids'* within the headlines of articles published within the selected newspapers. The initial search returned 1199

For peer review only - http://bmjopen.bmj.com/site/about/guidelines.xhtml

articles, which were subsequently subjected to manual application of exclusion criteria, including: less than 50% of article content being relevant to childhood obesity; being a reader's letter; or being part of television guide section. Following exclusion, the final sample comprised 757 relevant articles.

Coding

Article content was coded quantitatively using a coding frame adapted from one initially developed by Hilton and colleagues (21). The adapted coding frame was developed to record media frames of childhood obesity in terms of definitions of the problem, mentions of specific biological, individual and societal drivers, and biological, individual and societal solutions (itemised in Table 2). In addition, the coding frame recorded: whether the article was published on the front page of the publication; the length of the article; and the tone of headline. Each headline was coded as alarmist, neutral or reassuring. This code was based on the coders' interpretations of the editorial intent of the language used, mindful of the distinction between a headline being 'alarmist' and 'alarming'. Therefore, headlines that communicated potentially alarming news were not coded as 'alarmist' unless the coder judged the language in the headline to have been chosen specifically to provoke alarm in the reader. Coding was performed by AN and CP, and 10% of articles were double-coded blind to allow inter-rater agreement to be calculated. Cohen's kappa values for agreement on individual codes are listed in Table 2. The threshold for acceptable agreement was set at 0.61 (defined by Landis and Koch as 'substantial' or better agreement (42)), and three codes were removed due to insufficient agreement: dieting (such as fad diets) as a driver of childhood obesity; normalisation of obesity as a driver of childhood obesity; and technological developments as a driver of childhood obesity.

Analysis

Statistical analysis was performed in STATA. Statistical procedures included: basic descriptive statistics; Cohen's kappa test of inter-rater agreement; χ^2 tests of relationships between headline tone, market and political alignment; linear regression of relationships between publication year and

For peer review only - http://bmjopen.bmj.com/site/about/guidelines.xhtml

mentions of different categories of drivers and solutions; and multiple logistic regression of relationships between political alignment and individual aspects of framing. The multiple logistic regressions were adjusted by publication market because the markets represented were not distributed evenly by political alignment (as is the case in the UK newspaper industry), and previous research has identified significant variation in health news coverage by publication market (e.g. 21, 43, 44).

Comparative analysis

Data from Hilton and colleagues' previous study on representations of general (not childhood-specific) obesity in the UK media were also analysed which had been collected and described fully elsewhere(21) to enable comparison of newspaper representations of obesity in children with obesity in adults, and obesity coverage more generally. This direct comparison was enabled by the intentional similarity of the methods of data collection, coding and analysis in the two studies.

Patient and Public Involvement

Patient/public participants were not involved in this study.

Results

Sample characteristics

Table 1 summarises the political alignment and market of each publication in the sample, in addition to the frequency of articles and front-page articles within those publications, and the variation in word count within those articles. A total of 757 articles relevant to childhood obesity were identified within the selected six publications (five of which were combined with their corresponding Sunday counterparts). The frequency of coverage of childhood obesity varied between publications, ranging from the *Independent*& *Independent on Sunday* publishing 61 relevant articles, none of which were on front pages, to the *Mirror* & *Sunday Mirror*, which published 198 relevant articles, including two

For peer review only - http://bmjopen.bmj.com/site/about/guidelines.xhtml

front-page articles. The Daily Telegraph & Sunday Telegraph afforded the issue the greatest prominence, featuring it on their front pages nine times.

The changing frequency of relevant articles within the sample between 1996 and 2014 are illustrated in Figure 1, both overall and within each political alignment. The total number of relevant articles per year rose steadily from 2 in 1996 to a high of 82 in 2008, before declining to 40 in 2010, and finally rising again to 69 articles in 2014. The peak from 2006-08 was contemporaneous with the publication of the UK Government's Foresight project report on reducing obesity(8)and its corresponding mid-term and one-year reviews.

[Insert Figure 1. Frequency of articles by year]

Headline tone

Each article's headline was coded as either neutral (n=567, 74.9%), alarmist (n=165, 21.8%) or reassuring (n=22, 2.9%) in tone. Alarmist headlines outnumbered reassuring headlines within each publication and within each year, excluding 2001, in which three articles had reassuring headlines, compared to two alarmist headlines. *The Daily Mail* & *Mail on Sunday* exhibited both the highest proportion of alarmist headlines (n=43, 32.3%) and reassuring headlines (n=8, 6.0%). Headline tone varied significantly by publication market ($\chi^2(4)=28.6$, $p<0.001$), with alarmist headlines most common in tabloid publications (n=85, 51.5%). Headline tone did not vary significantly by political alignment ($\chi^2(2)=0.7$, $p<0.698$).

[Insert Table 1. Summary of article characteristics]

Definitions of the problem of childhood obesity

Table 2 illustrates the frequencies of articles mentioning specific problem definitions, drivers and solutions related to childhood obesity, and Table 3 illustrates the extent to which publications' political alignment predicted mentions of specific definitions. More than half of articles quantified

For peer review only - http://bmjopen.bmj.com/site/about/guidelines.xhtml

childhood obesity prevalence within the UK (n=413 54.6%), and a similar proportion described obesity prevalence as rising, or having risen (n=389, 51.4%). Centre-right-aligned publications mentioned increasing prevalence significantly less frequently than centre-left publications (OR:0.59; p=0.001). Eighty (10.6%) articles quantified the prevalence of obesity outside of the UK. Approximately half of articles specifically described obesity as a health risk (n=397, 52.4%), and 102 (13.5%) described it as a burden to the National Health Service, and each of these themes were more frequent in centre-left publications (OR:0.35, p=0.010; OR:0.50, p=0.008).Childhood obesity was characterised as an economic burden to society in 74 (9.8%) articles, and significantly more so in centre-left publications (OR:0.35, p=0.010).

[Insert Table 2. Frequency of mentions of problem definitions, drivers, and categories of solutions]

[Insert Table 3. Likelihood of centre-right-aligned publications mentioning definitions of obesity]

Few articles (n=23, 3.0%) characterised obesity as a cosmetic problem. Twice as many articles mentioned childhood obesity in relation to women and/or girls (n=112, 14.8%) as men and/or boys (n=56, 7.4%), and men and/or boys were more likely to be mentioned in centre-left publication than centre-right publications, after adjusting for market (OR:0.43, p=0.020).

Presentations of potential drivers of, and solutions to, childhood obesity

Mentions of specific drivers of childhood obesity were coded and categorised as either individual (n=453, 59.8%), societal (n=214, 28.3%) or biological/genetic (n=70, 9.2%) drivers (Table 2). Societal drivers were mentioned more frequently in centre-left publications (OR:0.69, p=0.046).Frequently-mentioned individual drivers included parenting (n=246, 32.5%), diet (n=235, 31.0%) and insufficient exercise (n=224, 29.6%), while societal drivers included an abundance of unhealthy food (n=129, 17.0%), marketing (n=90, 11.9%) and insufficient health services or facilities (n=53, 7.0%).

In addition to drivers, mentions of potential solutions to childhood obesity were coded into three corresponding categories: individual (n=276, 36.5%), societal (n=214, 28.3%) and biological (n=52,

For peer review only - http://bmjopen.bmj.com/site/about/guidelines.xhtml

6.9%) (Table 2).Table 4 illustrates the extent to which publications' political alignment predicted mentions of specific drivers and solutions. Centre-left publications were more likely to mention societal drivers (OR:0.69, p=0.046) and societal solutions (OR:0.54, p=0.046). Specific drivers that centre-left publications were more likely to mention included diet (OR:0.65, p=0.018), insufficient exercise (OR:0.67, p=0.032).and marketing(OR:0.55, 0.030).

[Insert Table 4. Likelihood of centre-right-aligned publications mentioning categories of driver and solution]

Time trends in presentation of drivers and solutions

Time trends in mentioning each category were analysed. Mentions of individual drivers (coefficient -0.068, p<0.001), individual solutions (coefficient -0.037, p=0.041), societal drivers (coefficient -0.097, p<0.001) and societal solutions (coefficient -0.044, p=0.012) each decreased significantly between 1996 and 2014. Neither biological/genetic drivers (coefficient -0.014, p=0.637) nor biological solutions (coefficient -0.020, p=0.558) varied significantly across the sample period.

Figure 2 illustrates the trends in individual and societal drivers and solutions. Individual drivers were mentioned particularly frequently (82-100%) between 1998 and 2000, before declining to between 46% and 67% of articles between 2004 and 2014. Mentions of individual solutions peaked at 83% in 2000, and subsequently declined, comprising 25-38% articles between 2007 and 2014. Mentions of societal drivers peaked at 67% in 2000, followed by a lower peak of 61% in 2002 and a subsequent lengthy decline to a low of 8% in 2012. Mentions of societal solutions exhibited a less linear decline than other categories, with peaks in 1998 (73%), 2004 (71%) and 2008 (66%), interspersed with declines. Notably, societal solutions were more commonly mentioned than individual solutions from 2007-2014.

For peer review only - http://bmjopen.bmj.com/site/about/guidelines.xhtml

[Insert Figure 2. Trends in individual and societal drivers and solutions]

Representations of childhood obesity in comparison to adult and general population obesity

The data collected for this study were compared with data collected in Hilton and colleagues' 2012 study of newspaper representations of obesity in the general population (21).Supporting information Figure S1 illustrates the yearly frequency of articles in each study's main sample, as well as a subsample of the previous study's data that excludes all articles that mentioned children. This represents a means of comparing representations of childhood obesity with representations of adult and non-age-specific obesity. Table S1 suggests that childhood obesity received less newspaper coverage than adult obesity in every year covered by the two datasets, with the exception of 1999. The longer time period represented in the current study suggests that the decrease in publication frequency in 2008-2010 observed in the previous study (21)did not continue in subsequent years, at least on the topic childhood obesity.

Supporting information Figure S2 illustrates the frequency of coverage of the two categories of driver and solution, individual and societal, within the present and prior sample(21). Comparison of the data indicates that coverage of childhood obesity was characterised by greater focus on individual drivers and societal solutions than coverage of adult obesity, while coverage of societal drivers and individual solutions was relatively similar.

Discussion

By systematically analysing the content of 757 articles, we arrived at several key findings related to UK national newspapers' representations of childhood obesity. Coverage of the issue grew steadily from 1996 to 2008, followed by a period of relatively infrequent coverage. Childhood obesity was predominantly characterised as driven by individual-level factors, particularly parenting, dietary

For peer review only - http://bmjopen.bmj.com/site/about/guidelines.xhtml

For peer review only

behaviours and inactivity, though societal drivers such as marketing were also identified. Similarly, there was greater focus on individual-level solutions than societal-level solutions. Societal constructions of the drivers of, and solutions to, obesity, were significantly more frequent within centre-left publications than centre-right. Analysis of time trends provided evidence of a small shift towards societal conceptualisations, with mentions of social solutions outnumbering individual solutions throughout the latter half of the sample period. Across the 19-year period studied, alarmist headlines greatly outnumbered reassuring headlines, particularly in tabloid publications, and more frequently in centre-right publications. Childhood obesity was frequently defined as a health risk in approximately half of articles, and was associated with females substantially more frequently than males, but more nuanced coding of gender-representation in these articles is required.

Centre-left publications' greater focus on societal constructions of the causes of, and solutions to childhood obesity, and on the societal and health service burdens of childhood obesity, are in line with the communal and individual framings associated with left- and right-wing political ideologies. Entman describes the core process of building frames as *"[selecting] some aspects of a perceived reality and [making] them more salient"*(16), and this process is evident in UK newspaper representations of childhood obesity, with centre-left publications building frames that incorporate societal aspects of the childhood obesity problem, while centre-right publications omit them.

This research comprised a systematic analysis of a large sample of nineteen years of UK national newspaper coverage, facilitating statistical understandings of media frames of childhood obesity, including definitions, drivers and solutions. However, the research is subject to some limitations. Using the *Nexis* database facilitates the systematic sampling of relevant newspaper content, but necessitates relying on the integrity of the database; as such, it is possible that some trends identified may be artefacts of inconsistencies in the database. The method allowed quantitative analysis of a media frames across a large sample, but not the nuanced analysis of specific aspects of framing that qualitative analysis would permit. The coding frame was extensive, but subject to

For peer review only - http://bmjopen.bmj.com/site/about/guidelines.xhtml

certain limitations. Coding did record the types of issues discussed by each articles, which may have been valuable given the variety of different perspectives from which the issue may be viewed. Further, while mentions of males and females in relation to obesity in children were coded, coding did not differentiate between mentions of boys with obesity, girls with obesity, male parents and female parents. Given the frequently gendered nature of societal discourse about obesity, future research may benefit from analysing gendered representations of both children and parents within news coverage of childhood obesity. Additionally, future research may benefit from widening the search scope from childhood obesity to also cover childhood overweight. Further limitations of the research stem from decisions made about the type of content analysed. The sole focus on article text was at the cost of analysing images, which have been found to be an important aspect of media representations of obesity (23, 31, 45, 46). Further, the focus on newspaper content was at the expense of data from other news sources, such as television and online news. We argue that our focus on the evolution of the debate over time is not well suited to the rapidly-changing online news environment, but incorporating other news sources could be valuable as representations of childhood obesity have been found to vary by medium in the US(34). Finally, while links between media representations and public perceptions are well established, content analysis can only describe content, not determine how that content is received by audiences.

This research built upon prior research examining media framing of general obesity (21) by extending the time period covered, taking a sole focus on childhood obesity, and comparing coverage of childhood obesity to that of obesity in general. As would be expected, the growth in coverage of childhood obesity from 1996 to 2008 identified in our prior research(21) was replicated in the present research, but it was found that the rise did not continue beyond 2008. As mentioned above, observed trends in data may be artefacts of inconsistencies in the *Nexis* database, but in the case of observed trends in overall article frequency, these are unlikely to be due to systematic inconsistencies in the database because the peaks and troughs in reporting were not uniform between publications, and the substantial reductions in article frequency cannot be explained by

For peer review only - http://bmjopen.bmj.com/site/about/guidelines.xhtml

gaps in specific publications' archives .One potential explanation for a reduction in coverage subsequent to 2008 is that coverage of childhood obesity in 2007-8 was elevated due to dissemination of, and activities related to, the UK Government's Foresight report *Reducing obesity: future choices*, published in October 2007(8).Further research might investigate whether the increase in article frequency in the final year of the study period is indicative of a prolonged rise in coverage beyond 2014. Both the original study by Hilton and colleagues and the present study present some evidence of a shift away from a focus on individual constructions of drivers and solutions across their respective time periods. However, comparison of the two pieces of research suggests that, in comparison to general obesity, framings of childhood obesity have a greater tendency to attribute responsibility to individuals. The disproportionate individual-level framing of childhood obesity might be explained by the presence of parents as mediators between children and public policy. While children are vulnerable to societal and environmental pressures, and are often publicly viewed as deserving of legislative protection(47, 48, 49), public discourse around childhood obesity may attribute greater individual responsibility to parents(50). Hawkins and Linvill found that US news frequently identifies parents as both responsible for, and responsible for addressing, children's obesity, and conclude that this framing represents an obstacle to stimulating demand for a public policy response to the problem (35). Boero's qualitative analysis of US media representations of childhood obesity identifies parents, and particularly mothers, as being 'under fire' for failing to foster healthy behaviours in their children(28). Unlike in debates around unhealthy phenomena such as exposure to second-hand smoke, in which an adult lifestyle product may be perceived as unfairly invading children's spaces, feeding children occupies a complex position of being nurturing and essential, while also being a potential source of long-term health harms(50).

The growth and decline in overall coverage of childhood obesity identified in the present study mirrored that found in Barry and colleagues'(34) content analysis of US television and print news coverage of childhood obesity suggesting that, despite locally-relevant policy events, trends in coverage of childhood obesity may follow transnational patterns. Barry and colleagues (34) suggest

For peer review only - http://bmjopen.bmj.com/site/about/guidelines.xhtml

that the decline in coverage may be an example of Downs'(51) "issue attention cycle", in which public attention to a specific issue will inevitably decline regardless of whether that issue reaches any conclusion. However, one area where our findings depart from those of Barry and colleagues (34) is in individual and structural causes of childhood obesity, which they found to be equally frequent within the newspaper articles in the their sample.

For media content to drive public appetite for policy solutions to childhood obesity, media must both raise perceptions of the issue, through heightened coverage, and frame the issue as one demanding societal level, rather than solely individual-level, solutions. Our research demonstrates that, while the salience of childhood obesity in UK national newspapers rose steadily from 1996-2008, that level of attention was not maintained subsequent to 2008, although there is reason to suggest that this may change in 2017/18 with media coverage of the incoming levy on sugar sweetened beverages in the UK. While this faltering frequency of reporting may be undesirable for raising public consciousness, our analysis suggests that the frames constructed within those later years were characterised by a predominance of social solutions over individual solutions, which, if internalised by audiences, may stimulate public appetites for engaging the problem at the public policy level. Notably, this shift from individual to social framing occurred despite the well-documented complications caused by parents' roles as mediators between public policy and children's health behaviours. Taking these key findings into account, this study supports a mixed view of UK media framing of childhood obesity, in which positive changes in framing may be undermined by a decrease in salience. Those advocating for public policy responses to childhood obesity may seek to raise the issue's media profile, while continuing to promote social framings.

For peer review only - http://bmjopen.bmj.com/site/about/guidelines.xhtml

Author Statement

SH, CP and AN: study planning and conceptualisation; AN and CP: data coding; CP and AN: data analysis; AN and CP: drafting manuscript; SH: critical review of the manuscript.

Data Statement

Data were accessed from the Nexis newspaper database at https://www.nexis.com

For peer review only

For peer review only - http://bmjopen.bmj.com/site/about/guidelines.xhtml

TABLES:

Table 1. Summary of article characteristics

Publication	Political alignment	Market	All articles		Front-page articles		Word count		
			n	%*	n	%**	1st quartile	Median	3rd quartile
Guardian & Observer	Centre-left	Quality	109	14.4	5	4.6	457	680	907
Independent & Independent on Sunday	Centre-left	Quality	61	8.1	0	-	247	474	690
Mirror & Sunday Mirror	Centre-left	Tabloid	198	26.2	2	1.0	121	219	459
Daily Telegraph & Sunday Telegraph	Centre-right	Quality	107	14.1	9	8.4	182	346	502
Daily Mail & Mail on Sunday	Centre-right	Middle-market	134	17.7	6	4.5	263	438	672
Sun	Centre-right	Tabloid	148	19.6	0	-	98	195	337
		**Total**	**757**	**100.0**	**22**	**2.9**	**151**	**325**	**595**

*percentage within whole sample
**percentage of front-page articles within publication

For peer review only - http://bmjopen.bmj.com/site/about/guidelines.xhtml

Table 2. Frequency of mentions of problem definitions, drivers, and categories of solutions

Theme	Total (n=757) n	%	Inter-rater agreement*
**Problem definitions**			
Quantifies obesity prevalence within the UK	413	54.6	0.834
Quantifies obesity prevalence elsewhere	80	10.6	0.814
Mentions increase in obesity rates	389	51.4	0.940
Mentions obesity as a risk to health	397	52.4	0.893
Mentions obesity as a cosmetic problem	23	3.0	0.850
Mentions obesity as a burden to NHS	102	13.5	0.814
Mentions obesity as an economic burden to society	32	4.2	0.630
Mentions socio-economic and geographical differences	74	9.8	0.706
Mentions women and/or girls	112	14.8	0.706
Mentions men and/or boys	56	7.4	0.706
Obesity is not a problem, over-hyped etc.	93	12.3	0.850
Mentions discrimination, bullying or stigmatisation	70	9.2	1.000
**Drivers of obesity**			
Overall drivers			
Any drivers mentioned	522	69.0	n/a**
Any biological/genetic driver mentioned	70	9.2	n/a**
Any individual driver mentioned	453	59.8	n/a**
Any societal driver mentioned	214	28.3	n/a**
Individual drivers			
Mentions poor diet, overeating	235	31.0	0.857
Mentions poor self-control, willpower or choices	60	7.9	0.680
Mentions insufficient exercise, sedentary lifestyle	224	29.6	0.919
Mentions parenting shortcomings	246	32.5	0.939
Societal drivers			
Mentions an abundance of processed/fast food	129	17.0	0.752
Mentions a lack of health services or facilities	53	7.0	0.945
Mentions food/drink advertising and promotions	90	11.9	1.000
**Solutions to obesity**			
Any solution mentioned	538	71.1	n/a**
Individual solution mentioned	276	36.5	0.920
Societal solution mentioned	214	28.3	0.839
Biological solution mentioned	52	6.9	1.000

*Cohen's kappa test of inter-rater agreement.

**Agreement was not calculated for these variables as they were computed from other, manually-coded variables

For peer review only - http://bmjopen.bmj.com/site/about/guidelines.xhtml

Table 3. Likelihood of centre-right-aligned publications mentioning definitions of obesity

	Unadjusted			Adjusted*		
	OR	95% CI	P-value	OR	95% CI	P-value
**Problem definitions**						
Quantifies obesity prevalence within the UK						
	0.97	0.73-1.30	0.858	0.92	0.67-1.27	0.608
Quantifies obesity prevalence elsewhere						
	0.63	0.40-1.01	0.057	0.59	0.34-1.03	0.065
Mentions increase in obesity rates						
	0.70	0.52-0.93	**0.014**	0.59	0.42-0.81	**0.001**
Mentions obesity as a risk to health						
	1.02	0.77-1.36	0.885	0.88	0.64-1.22	0.456
Mentions obesity as a cosmetic problem						
	0.40	0.16-0.99	**0.048**	0.35	0.11-1.05	0.061
Mentions obesity as a burden to NHS						
	0.57	0.37-0.87	**0.009**	0.50	0.30-0.83	**0.008**
Mentions obesity as an economic burden to society						
	0.36	0.19-0.70	**0.003**	0.35	0.16-0.78	**0.010**
Mentions socio-economic and geographical differences						
	0.62	0.38-1.00	0.051	0.85	0.51-1.43	0.547
Mentions women and/or girls						
	0.86	0.58-1.29	0.467	0.77	0.48-1.23	0.271
Mentions men and/or boys						
	0.59	0.34-1.03	0.062	0.43	0.22-0.88	**0.020**
Obesity is not a problem, over-hyped etc.						
	0.75	0.29-1.93	0.552	0.73	0.25-2.15	0.565
Mentions discrimination, bullying or stigmatisation						
	0.56	0.36-0.87	**0.010**	0.44	0.25-0.76	**0.003**

*Adjusted for publication market

For peer review only

Table 4. Likelihood of centre-right-aligned publications mentioning categories of driver and solution

	Unadjusted			Adjusted*		
	OR	95% CI	P-value	OR	95% CI	P-value
**Drivers of obesity**						
Overall drivers						
Any drivers mentioned						
	0.90	0.66-1.23	0.505	0.78	0.56-1.10	0.162
Any biological/genetic driver mentioned						
	0.73	0.45-1.20	0.214	0.85	0.49-1.46	0.557
Any individual driver mentioned						
	1.00	0.75-1.34	0.974	0.84	0.61-1.16	0.292
Any societal driver mentioned						
	0.62	0.45-0.86	**0.004**	0.69	0.48-0.99	**0.046**
Individual drivers						
Mentions poor diet, overeating						
	0.73	0.54-0.99	**0.045**	0.65	0.46-0.93	**0.018**
Mentions poor self-control, willpower or choices						
	0.61	0.35-1.04	0.068	0.71	0.39-1.28	0.255
Mentions insufficient exercise, sedentary lifestyle						
	0.75	0.55-1.03	0.077	0.67	0.47-0.97	**0.032**
Mentions parenting shortcomings						
	1.14	0.84-1.55	0.386	1.08	0.77-1.52	0.660
Societal drivers						
Mentions an abundance of processed/fast food						
	0.61	0.41-0.89	**0.011**	0.73	0.48-1.12	0.153
Mentions a lack of health services or facilities						
	0.90	0.52-1.58	0.725	0.87	0.46-1.65	0.671
Mentions food/drink advertising and promotions						
	0.56	0.36-0.88	**0.012**	0.55	0.32-0.94	**0.030**
**Solutions to obesity**						
Biological						
	0.73	0.42-1.29	0.286	0.54	0.26-1.09	0.087
Individual						
	0.90	0.67-1.20	0.464	0.90	0.64-1.25	0.527
Societal						
	0.62	0.46-0.83	**0.001**	0.54	0.39-0.75	**0.000**

*Adjusted for publication market

For peer review only - http://bmjopen.bmj.com/site/about/guidelines.xhtml

For peer review only

For peer review only - http://bmjopen.bmj.com/site/about/guidelines.xhtml

References

1. Ebbeling CB, Pawlak DB, Ludwig DS. Childhood obesity: public-health crisis, common sense cure. *Lancet* 2002;**360:** 473-482.

2. Ng M, Fleming T, Robinson M, Thomson B, Graetz N, Margono C*, et al.* Global, regional, and national prevalence of overweight and obesity in children and adults during 1980–2013: a systematic analysis for the Global Burden of Disease Study 2013. *Lancet* 2014;**384:** 766-781.

3. NHS Digital. Statistics on Obesity, Physical Activity and Diet. 2017.

4. Scottish Government. The Scottish Health Survey: 2015 edition. Scottish Government: Edinburgh, 2015.

5. Simmonds M, Llewellyn A, Owen CG, Woolacott N. Predicting adult obesity from childhood obesity: a systematic review and meta-analysis. *Obesity Reviews* 2016;**17:** 95-107.

6. HM Government. Childhood obesity: a plan for action. 2017.

7. Scottish Government. Increase the proportion of healthy weight children 2016 [4th August 2017]. Available from: http://www.gov.scot/About/Performance/scotPerforms/indicator/healthyweight.

8. Butland B, Jebb S, Kopelman P, McPherson K, Thomas S, Mardell J*, et al.* Foresight. Tackling Obesities: Future Choices - Project report. Government Office for Science: London, 2007.

9. Friedman DA. Public health regulation and the limits of paternalism. *Conn L Rev* 2013;**46:** 1687.

10. Swinburn BA, Sacks G, Hall KD, McPherson K, Finegood DT, Moodie ML*, et al.* The global obesity pandemic: shaped by global drivers and local environments. *Lancet* 2011;**378:** 804-814.

11. Swinburn BA. Obesity prevention: the role of policies, laws and regulations. *Australia and New Zealand health policy* 2008;**5:** 12.

12. Hilbert A, Rief W, Braehler E. What determines public support of obesity prevention? *J Epidemiol Community Health* 2007;**61:** 585.

13. Evans WD, Finkelstein EA, Kamerow DB, Renaud JM. Public perceptions of childhood obesity. *Am J Prev Med* 2005;**28:** 26-32.

For peer review only - http://bmjopen.bmj.com/site/about/guidelines.xhtml

14. McCombs ME, Shaw DL. The agenda-setting function of mass media. *Public Opin Q* 1972;**36:** 176-187.

15. Scheufele DA, Tewksbury D. Framing, agenda setting, and priming: The evolution of three media effects models. *J Commun* 2007;**57:** 9-20.

16. Entman RM. Framing: Toward clarification of a fractured paradigm. *J Commun* 1993;**43:** 51-58.

17. Barry CL, Brescoll VL, Gollust SE. Framing Childhood Obesity: How Individualizing the Problem Affects Public Support for Prevention. *Polit Psychol* 2013**:** n/a-n/a.

18. Gollust SE, Niederdeppe J, Barry CL. Framing the Consequences of Childhood Obesity to Increase Public Support for Obesity Prevention Policy. *Am J Public Health* 2013;**103:** e96-e102.

19. Barry CL, Brescoll VL, Brownell KD, Schlesinger M. Obesity Metaphors: How Beliefs about the Causes of Obesity Affect Support for Public Policy. *Milbank Q* 2009;**87:** 7-47.

20. Boyce T. The media and obesity. *Obesity Reviews* 2007;**8:** 201-205.

21. Hilton S, Patterson C, Teyhan A. Escalating coverage of obesity in UK newspapers: the evolution and framing of the "obesity epidemic" from 1996 to 2010. *Obes* 2012;**20:** 1688-1695.

22. Hilbert A, Ried J. Obesity in Print: An Analysis of Daily Newspapers. *Obesity Facts* 2009;**2:** 46-51.

23. Patterson C, Hilton S. Normalisation and Stigmatisation of Obesity in UK Newspapers: a Visual Content Analysis. *The Open Obesity Journal* 2013;**5:** 82-91.

24. Flint SW, Hudson J, Lavallee D. The portrayal of obesity in UK national newspapers. *Stigma and Health* 2016;**1:** 16.

25. Islam S, Fitzgerald L. Indigenous obesity in the news: a media analysis of news representation of obesity in Australia's Indigenous population. *BMC obesity* 2016;**3:** 30.

26. Atanasova D. Obesity frames in British and German online newspapers, 2009-2011: Department of Media and Communication; 2015.

27. Bonfiglioli C. Overweight and Obesity in TV News. *Values and Choices in Television Discourse*. Springer, 2015, pp 87-108.

For peer review only

For peer review only - http://bmjopen.bmj.com/site/about/guidelines.xhtml

28. Boero N. All the news that's fat to print: the American "obesity epidemic" and the media. *Qualitative Sociology* 2007;**30:** 41-60.

29. Lawrence RG. Framing obesity: The evolution of news discourse on a public health issue. *Harvard International Journal of Press/Politics* 2004;**9:** 56-75.

30. Kim S-H, Willis LA. Talking about Obesity: News Framing of Who Is Responsible for Causing and Fixing the Problem. *Journal of Health Communication* 2007;**12:** 359-376.

31. Gollust SE, Eboh I, Barry CL. Picturing obesity: Analyzing the social epidemiology of obesity conveyed through US news media images. *Soc Sci Med* 2012;**74:** 1544-1551.

32. Jeong M, Gilmore JS, Bleakley A, Jordan A. Local news media framing of obesity in the context of a sugar-sweetened beverage reduction media campaign. *Journal of nutrition education and behavior* 2014;**46:** 583-588.

33. Varava K. Visual framing, stigmatization, race, and obesity: Examining television news presentation of stories about obesity and stories about health. *Communication Studies* 2016;**67:** 509-529.

34. Barry CL, Jarlenski M, Grob R, Schlesinger M, Gollust SE. News media framing of childhood obesity in the United States from 2000 to 2009. *Pediatrics* 2011;**128:** 132-145.

35. Hawkins KW, Linvill DL. Public health framing of news regarding childhood obesity in the United States. *Health communication* 2010;**25:** 709-717.

36. Kalin SR, Fung TT. Comparison of child obesity prevention and control content in mainstream and Spanish-language US parenting magazines. *Journal of the Academy of Nutrition and Dietetics* 2013;**113:** 133-140.

37. Bastian A. Representations of childhood obesity in Australian newsprint media and academic literature. *Aust N Z J Public Health* 2011;**35:** 135-139.

38. Maher J, Fraser S, Wright J. Framing the mother: childhood obesity, maternal responsibility and care. *J Gend Stud* 2010;**19:** 233-247.

39. Boero N. Obesity in the media: Social science weighs in. *Critical Public Health* 2013;**23:** 371-380.

40. Douglas F, Clark J, Craig L, Campbell J, McNeill G. "It'sa balance of just getting things right": mothers' views about pre-school childhood obesity and obesity prevention in Scotland. *BMC public health* 2014;**14:** 1009.

For peer review only - http://bmjopen.bmj.com/site/about/guidelines.xhtml

41. Press Gazette. National newspaper print ABCs for Jan 2017: Times and Observer both boost print sales year on year 2017 [cited 2017 2nd August 2017]. Available from: http://www.pressgazette.co.uk/national-newspaper-print-abcs-for-jan-2017-observer-up-year-on-year-the-sun-is-fastest-riser-month-on-month/.

42. Landis JR, Koch GG. The measurement of observer agreement for categorical data *Biometrics* 1977;**33:** 159-174.

43. Patterson C, Semple S, Wood K, Duffy S, Hilton S. A quantitative content analysis of UK newsprint coverage of proposed legislation to prohibit smoking in private vehicles carrying children. *BMC Public Health* 2015;**15:** 760.

44. Patterson C, Katikireddi SV, Wood K, Hilton S. Representations of minimum unit pricing for alcohol in UK newspapers: a case study of a public health policy debate. *J Public Health* 2014;**37:** 40-49.

45. Heuer CA, McClure KJ, Puhl RM. Obesity stigma in online news: A visual content analysis. *Journal of Health Communication* 2011;**16:** 976-987.

46. McClure KJ, Puhl RM, Heuer CA. Obesity in the news: Do photographic images of obese persons influence antifat attitudes? *Journal of health communication* 2011;**16:** 359-371.

47. Schneider A, Ingram H. Social construction of target populations: Implications for politics and policy. *Am Polit Sci Rev* 1993**:** 334-347.

48. Freeman B, Chapman S, Storey P. Banning smoking in cars carrying children: an analytical history of a public health advocacy campaign. *Aust N Z J Public Health* 2008;**32:** 60-65.

49. Hilton S, Wood K, Bain J, Patterson C, Duffy S, Semple S. Newsprint coverage of smoking in cars carrying children: a case study of public and scientific opinion driving the policy debate. *BMC Public Health* 2014;**14:** 1116.

50. Schwartz MB, Puhl R. Childhood obesity: a societal problem to solve. *Obesity reviews* 2003;**4:** 57-71.

51. Downs A. Up and down with ecology: the “issue-attention cycle”. *The public* 1972.

For peer review only - http://bmjopen.bmj.com/site/about/guidelines.xhtml

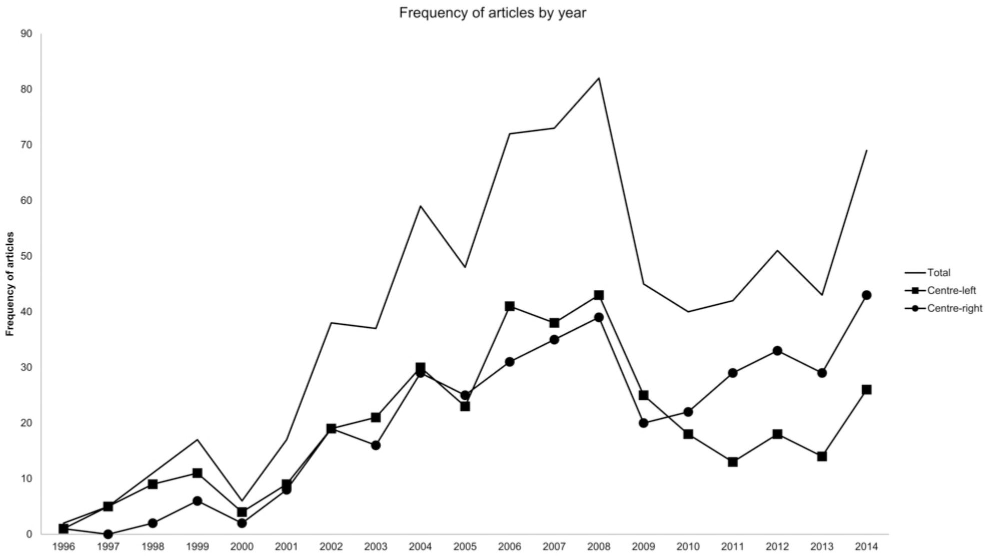

Figure 1. Frequency of articles by year

84x47mm (300 x 300 DPI)

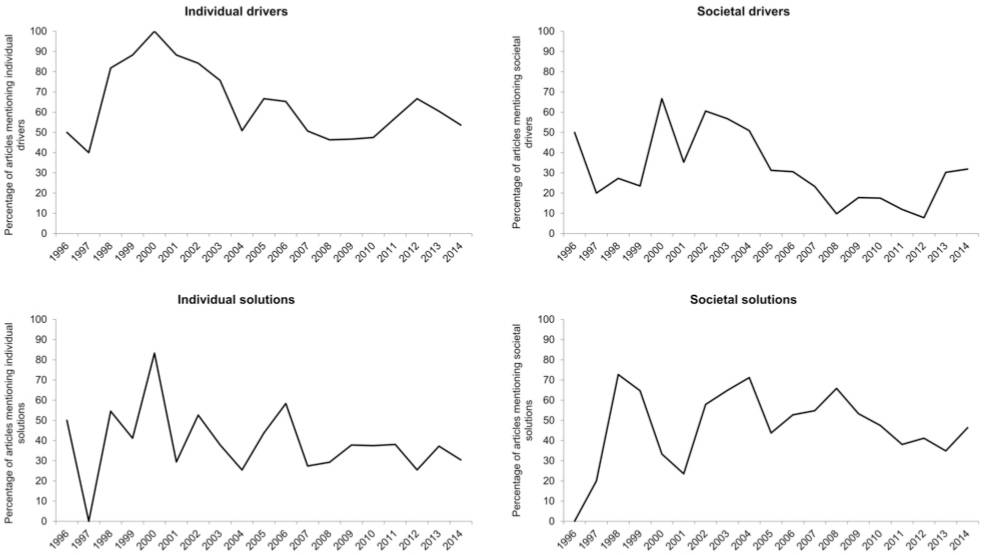

Figure 2. Trends in individual and societal drivers and solutions

84x47mm (300 x 300 DPI)

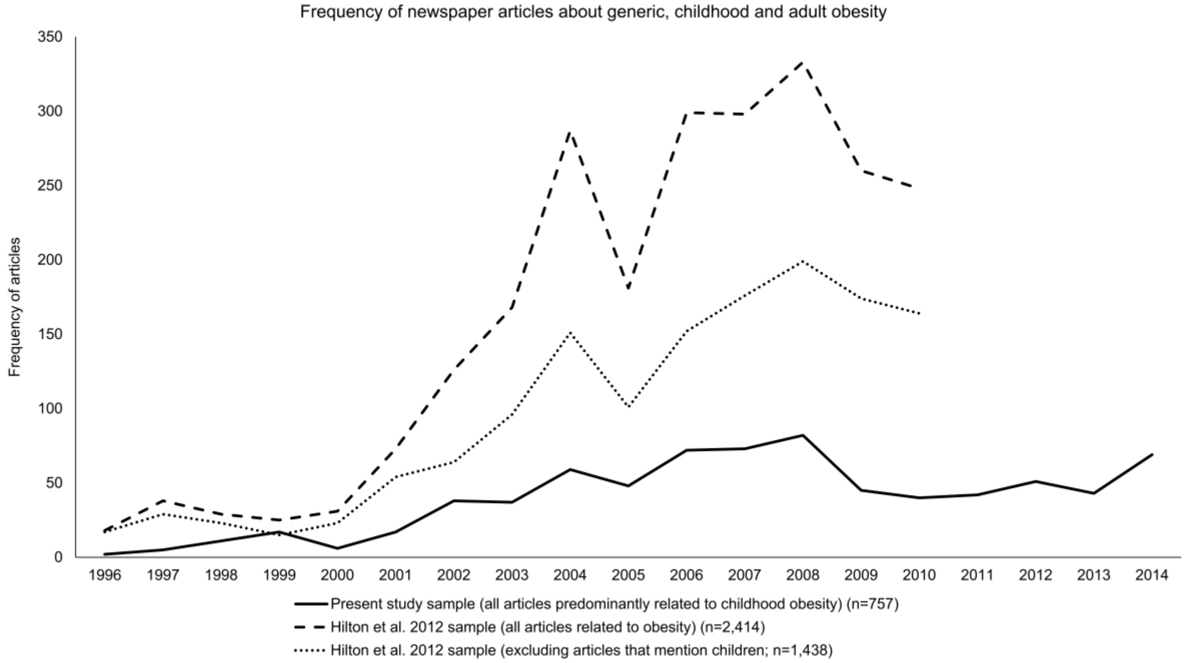
Frequency of newspaper articles about generic, childhood and adult obesity
Frequency of articles
Present study sample (all articles predominantly related to childhood obesity) (n=757)
Hilton et al. 2012 sample (all articles related to obesity) (n=2,414)
Hilton et al. 2012 sample (excluding articles that mention children; n=1,438)

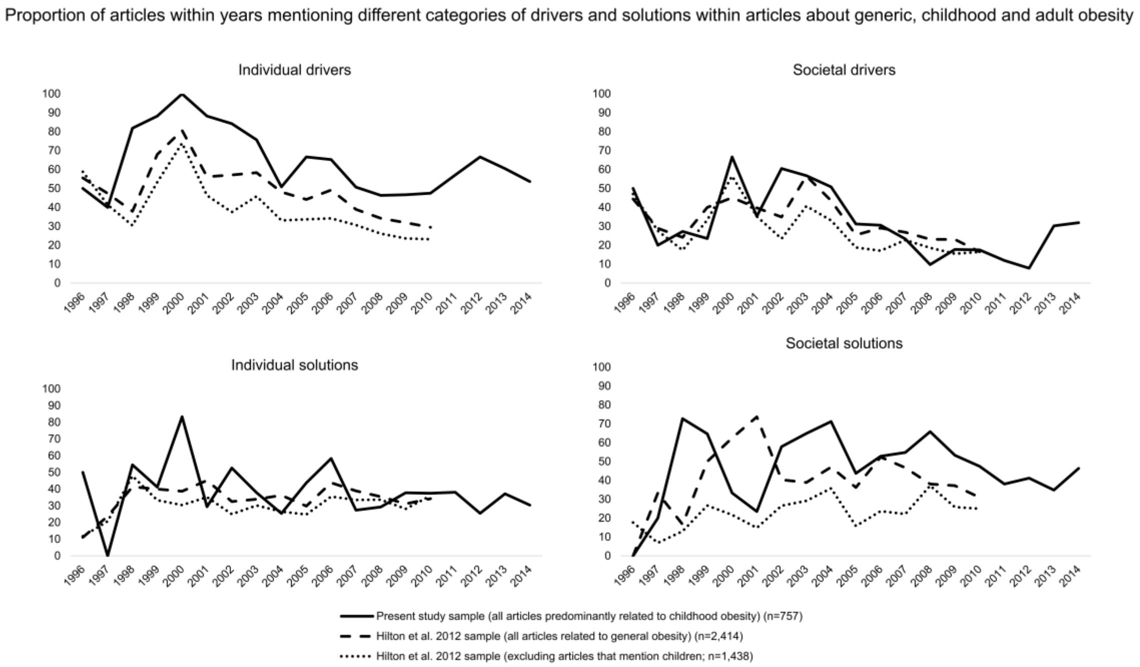
Proportion of articles within years mentioning different categories of drivers and solutions within articles about generic, childhood and adult obesity
Individual drivers
Societal drivers
Individual solutions
Societal solutions
Present study sample (all articles predominantly related to childhood obesity) (n=757)
Hilton et al. 2012 sample (all articles related to general obesity) (n=2,414)
Hilton et al. 2012 sample (excluding articles that mention children; n=1,438)

BMJ Open

Media framing of childhood obesity: a content analysis of UK newspapers from 1996-2014

Journal:	*BMJ Open*
Manuscript ID	bmjopen-2018-025646.R1
Article Type:	Research
Date Submitted by the Author:	07-Jan-2019
Complete List of Authors:	Nimegeer, Amy; Glasgow University, MRC/CSO Social and Public Health Sciences<br>Patterson, Chris; University of Glasgow, MRC/CSO Social & Public Health Sciences Unit<br>Hilton , Shona; University of Glasgow, MRC/CSO Social & Public Health Sciences Unit
**Primary Subject Heading**:	Communication
Secondary Subject Heading:	Sociology, Public health
Keywords:	Health policy < HEALTH SERVICES ADMINISTRATION & MANAGEMENT, MEDICAL JOURNALISM, Community child health < PAEDIATRICS, PAEDIATRICS, PUBLIC HEALTH, STATISTICS & RESEARCH METHODS

SCHOLARONE™
Manuscripts

TITLE: Media framing of childhood obesity: a content analysis of UK newspapers from 1996-2014

AUTHORS: Amy Nimegeer[1], Chris Patterson[1], Shona Hilton[1]

AFFILIATION: [1]MRC/CSO Social and Public Health Sciences Unit, University of Glasgow, 200 Renfield Street, Glasgow, G2 3QB, United Kingdom

KEYWORDS: Obesity, Children, Media, content analysis

RUNNING TITLE: UK Media framing of childhood obesity

CONTACT INFORMATION:

Dr Amy Nimegeer, MRC/CSO Social and Public Health Sciences Unit, University of Glasgow, 200 Renfield Street, Glasgow, G2 3QB, United Kingdom

amy.nimegeer@glasgow.ac.uk

Emails for additional authors: chris.patterson@glasgow.ac.uk ; shona.hilton@glasgow.ac.uk

WORD COUNT (excluding cover page, abstract, references, tables, and figures): 4166

For peer review only

Abstract

Background: Media can influence public and policymakers' perceptions of causes of, and solutions to, public health issues through selective presentation and framing. Childhood obesity is a health issue with both individual- and societal-level drivers and solutions, but public opinion and mass media representations of obesity have typically focused on individual-level framings, at the cost of acknowledgement of a need for regulatory action.

Objective and Setting: To understand the salience and framing of childhood obesity across 19 years of UK national newspaper content.

Design and Outcome Measures: Quantitative content analysis of 757 articles about childhood obesity obtained from six daily and five Sunday newspapers. Articles were coded manually for definitions, drivers and potential solutions. Data were analysed statistically, including analysis of time trends and variations by political alignment of source.

Results: The frequency of articles grew from a low of two in 1996 to a peak of 82 in 2008, before declining to 40 in 2010. Individual-level drivers (59.8%) and solutions (36.5%) were mentioned more frequently than societal-level drivers (28.3%) and solutions (28.3%) across the sample, but societal solutions were mentioned more frequently during the final eight years, coinciding with a marked decline in yearly frequency of articles.

Conclusions: Increased focus on societal solutions aligns with public health goals, but coincided with a reduction in the issue's salience in the media. Those advocating public policy solutions to childhood obesity may benefit from seeking to raise the issue's media profile while continuing to promote structural conceptualisations of childhood obesity.

For peer review only - http://bmjopen.bmj.com/site/about/guidelines.xhtml

Article Summary

Strengths and Limitations of this Study

Strengths

- Methodology includes systematic analysis of a large sample of nineteen years of UK national newspaper coverage, facilitating statistical understandings of media frames of childhood obesity, including definitions, drivers and solutions
- Features robust manual coding and links to pre-existing dataset to strengthen analysis

Weaknesses

- Quantitative media content analysis is inherently less sensitive to nuance than qualitative analysis, and our analysis excluded some aspects of media content, such as images, which may influence readers' interpretations of the text they accompany
- Content analysis is a means of documenting what messages are presented by media, but cannot tell us how these messages are received by audiences

Funding Statement

AN, CP and SH's time for this research was funded by the Informing Healthy Public Policy programme (MC_UU_12017-15 and SPHSU15) of the MRC/CSO Social and Public Health Sciences Unit, University of Glasgow. The funding bodies had no role in the design, collection, analysis or interpretation of this study.

Competing Interests

SH, CP and AN declare no conflicts of interest.

Acknowledgements

For peer review only - http://bmjopen.bmj.com/site/about/guidelines.xhtml

The authors would like to thank Dr Jonathan Olsen of the MRC/CSO Social and Public Health Sciences Unit, University of Glasgow, for his advice regarding the conduct of multiple logistic regression analysis. We would also like to acknowledge the contribution of Dr Marie Löf and Dr Mark Daku to the design of this study.

For peer review only

For peer review only - http://bmjopen.bmj.com/site/about/guidelines.xhtml

Introduction

Childhood obesity has been described as an international epidemic due to its high prevalence and rapid growth in numerous countries.[1] Globally, 13.4% of girls and 12.9% of boys in Low Income Countries, and 22.6% of girls, and 23.8% of boys in High Income Countries (classified by the World Bank) were classified as overweight or obese in 2013.[2] In England, one fifth of children in Reception year (age 4-5), and one third in Year 6 (age 10-11), were classified as overweight or obese in 2015/2016.[3] In Scotland, 28% of children aged 2-15 were classified as 'at risk of' overweight or obesity in 2015 [4]. Childhood obesity has a broad range of short- and long-term health consequences,[1] tends to predict adolescent and adult obesity,[5] and is socioeconomically patterned.[3] For these reasons childhood obesity has been identified as a health priority for the UK and its devolved governments.[6 7]

Childhood obesity is a complex problem, with a complex set of drivers and potential solutions ranging from the individual to the environmental.[8] Ebbeling and colleagues[1] identify a wide range of causes, but argue that the problem *"can be primarily attributed to adverse environmental factors"*, and identify a need for *"straightforward, if politically difficult"* solutions spanning homes, schools, the built environment, health care, marketing, media and politics. This multi-level package of solutions echoes Friedman's assertion that a 'full-court press' targeting 'every dimension of the problem' is necessary.[9] However, while academia and public health are united on the need to target the obesogenic environment, Swinburn and colleagues[10] state that *"governments have largely abdicated the responsibility for addressing obesity to individuals, the private sector, and non-governmental organisations",*[10] potentially due to anticipated or actual resistance, not just from corporations, but also electorates.[9 11] Indeed, public opinion research conducted in the US[12] and Germany[13] suggests that, while publics are in favour of tackling childhood obesity, they demonstrate less enthusiasm for regulative environmental interventions such as taxation. Hilbert characterises the German population

For peer review only - http://bmjopen.bmj.com/site/about/guidelines.xhtml

as *"ready for obesity prevention"*, but in need of education about the definition, prevalence and causes of obesity.[12]

The media represent a key influence on public perceptions of health issues and policies, setting the public agenda by granting differing levels of prominence to different topics[14] and influencing how those issues are understood by building frames (focuses of attention) that include constructions of problems, affected groups, drivers and solutions.[15 16] The influence of framing is well established in relation to obesity. Researchers have used experimental designs to demonstrate that 'individualised' representations of childhood obesity tend to lead participants to assign greater blame to individuals and exhibit less support for environmental regulation,[17] and that different representations of the consequences of childhood obesity can influence participants' attitudes towards policies.[18] Similarly, Barry and colleagues demonstrated that people's perceptions of obesity (as communicated through agreement with metaphor-based descriptions of obesity) predict their support for public policy interventions, illustrating how, for example, framing obesity as being driven by industry manipulation may lead to increased support for a 'junk-food tax'.[19]

The media are frequently accused of contributing to obesity, particularly childhood obesity, through its associations with sedentary behaviour, advertising of unhealthy commodities, promotion of unrealistic body image, and other mechanisms.[20] Many researchers have studied media representations of obesity in general,[21-33] but relatively few have focused specifically on representations of childhood obesity, and these have been primarily in the US and Australia. Barry and colleagues[34] studied US print and television news framing of childhood obesity, observing that coverage of the issue grew between 2000 and 2009, and that individual-level behavioural solutions to obesity were dominant, particularly on television. Similarly, Hawkins and Linvill[35] studied US newspaper framing of childhood obesity over three discrete time periods in 1991, 2001 and 2006, and identified a predominant focus on individual-level factors (both individual children and their parents) in representations of both causes and solutions.

For peer review only - http://bmjopen.bmj.com/site/about/guidelines.xhtml

For peer review only

Bastian[36] analysed representations of childhood obesity in both Australian newspapers and academic literature in 2009, identifying predominantly individual framing within the media, compared to a social-structural framing in academic literature. Bastian[36] recommends that public health professionals work to redirect media attention towards structural drivers of childhood obesity. Maher and colleagues[37] analysed constructions of maternal responsibilities within Australian media coverage of childhood obesity, concluding that the dominant framing *"individualises maternal and child relationships rather than seeing mothering as embedded in broader social and economic structures"*, serving a neo-liberal agenda by diminishing the responsibility of wider society. This is consistent with the disproportionate focus on individual-level solutions identified by others.[34-36 38] While coverage of obesity in both adults and children appears to be characterised by individual-framing, it is notable that with adult obesity that individual responsibility is assigned to the person with obesity, while in childhood obesity that responsibility is predominantly assigned to parents, particularly mothers.[39 40] This distinction may complicate direct comparison between adult and child obesity, and the culturally-ingrained nature of the concept that parents (or mothers) are solely responsible for their children's healthcare may represent a discursive obstacle to attempts to assign environmental solutions to childhood obesity.

In addition to traditional news media, researchers have analysed representations of childhood obesity in non-news media and new media. For example, Kalin and Fung's[38] analysis of Spanish-language US parenting magazines' representations of childhood obesity prevention and control echoes studies of news media representations of obesity, identifying greater focus on parental behaviour-change than system-level solutions, and limited recognition of social contextual factors. In recognition of the growing importance of user-generated social content and discussion, researchers have increasingly analysed content about childhood obesity on social media platforms.[41 42] While these new forms of media content represent an important aspect of the changing media landscape, traditional media outlets remain influential; despite the precipitous decline of UK print newspaper circulation,[43] the online presences of these hegemonic print news brands largely dominate online news readership,[44]

For peer review only - http://bmjopen.bmj.com/site/about/guidelines.xhtml

and typically define or legitimise news agendas for social media discussion.[45 46] As such, traditional media remain a relevant subject for media analysis, particularly when studying how representations evolve over timeframes predating the ascendancy of new media.

The aim of this study is to further understandings of media representations of childhood obesity in the UK context, using an approach informed by media framing theory,[15 16] analysing definitions of the problem and constructions of drivers and solutions. This is important because, while childhood obesity in the UK shares many similarities with that of other countries, the UK context differs in terms of several elements including health service structure and media environment. The analysis will have dual foci: the evolution of coverage between 1996 and 2014, and the relative salience of individual and societal constructions of the drivers of, and potential solutions to, childhood obesity. To our knowledge, this research will be the first empirical analysis of UK media framing of childhood obesity. This paper comprises the UK portion of a multi-country research project, the other parts of which will be reported in separate papers.

Methods

The media content analysis methods used were predominantly based on Hilton and colleagues' prior study[21] of UK newspaper framing of obesity in the general population, adapted for this study's focus on childhood obesity. This paper reports UK data that was part of a wider study that examined childhood obesity media coverage in two other international contexts; Sweden and the United States. Although content analysis is often viewed as an objective, descriptive approach, we subscribe to Krippendorff's position that even the quantitative analysis of text is inherently an interpretive act, and researchers should therefore acknowledge the individual bias that can arise from that process, seeking to minimise that bias through research design, while also embracing how researchers' contextual understandings can enrich coding and analysis beyond the crude 'objective' counting of content.

For peer review only - http://bmjopen.bmj.com/site/about/guidelines.xhtml

Patient and Public Involvement

Due to the nature of this study, patients/public were not involved.

Sampling

A set of six daily newspapers and five Sunday newspapers with high circulation figures[47] and representing a variety of political alignments and markets (or 'genres') were chosen. Table 1 lists these publications and indicates their political alignments and the markets that they occupy. Markets were defined as tabloid (typically sensationalist and politically diverse, with predominantly working-class readerships), middle-market tabloid (centre-right content with predominantly older, middle-class readerships) and quality (serious tone with predominantly middle-class readerships), using a typology used in prior studies of UK newspaper content[21]. Political alignment was determined by cross-referencing data on: the political party endorsed by each publication at the 2017 UK general election;[48] readers' perceptions of newspapers' political alignment;[49] and the voting behaviours of each publications' readers in the 2015 UK general election. [50] A sample period of 1996 to 2014 was chosen to encompass the time period covered in prior research,[21] in addition to a further four years of coverage that was extended to align with the time period covered by the other countries in our wider study (which will be described fully in a separate publication).

Identifying relevant articles from the chosen publications involved an initial database search, followed by manual filtering of search results. The Nexis database was searched for the presence of both the term *'obesity' OR 'obese' OR 'fat'* and the term *'child' OR 'children' OR 'kid' OR kids'* within the headlines of articles published within the selected newspapers. Each chosen publication was archived comprehensively within the Nexis database, with the exceptions of the Daily Telegraph and Sunday Telegraph prior to October 2000 and November 2000, respectively. As such, reporting from those publications during the first five years of the sample period was not represented. The initial search returned 1199 articles, which were subsequently subjected to manual application of exclusion criteria, including: less than 50% of article content focussing on childhood obesity (i.e.

For peer review only - http://bmjopen.bmj.com/site/about/guidelines.xhtml

where more than half the article discussed another topic with only brief mention of childhood obesity); being a reader's letter; or being part of television guide section. Following exclusion, the final sample comprised 757 relevant articles.

Coding

Article content was coded quantitatively using a coding frame adapted from one initially developed by Hilton and colleagues.[21] The adapted coding frame was developed to record media frames of childhood obesity in terms of definitions of the problem, mentions of specific biological, individual and societal drivers, and biological, individual and societal solutions (itemised in Table 2). In addition, the coding frame recorded whether the article was published on the front page of the publication, and the length of the article in number of words. Articles were coded as relating to women/girls or men/boys if members of that gender were described as being specifically problematic in relation to childhood obesity (but not if rates for both genders were cited), or if the article profiled an individual of a specific gender. Coding was performed by AN and CP, and 10% of articles were double-coded blind to allow inter-rater agreement to be calculated. Cohen's kappa values for agreement on individual codes are listed in Table 2. The threshold for acceptable agreement was set at 0.61 (defined by Landis and Koch as 'substantial' or better agreement),[51] and three codes were removed due to insufficient agreement: dieting (such as fad diets) as a driver of childhood obesity; normalisation of obesity as a driver of childhood obesity; and technological developments as a driver of childhood obesity.

Analysis

Statistical analysis was performed in STATA. Statistical procedures included: basic descriptive statistics; Cohen's kappa test of inter-rater agreement; linear regression of relationships between publication year and mentions of different categories of drivers and solutions; and multiple logistic regression of relationships between political alignment and individual aspects of framing. The multiple logistic regressions were adjusted by publication market because the markets represented

For peer review only - http://bmjopen.bmj.com/site/about/guidelines.xhtml

were not distributed evenly by political alignment (as is the case in the UK newspaper industry), and previous research has identified significant variation in health news coverage by publication market.[21 52 53]

Comparative analysis

Data from Hilton and colleagues' previous study on representations of general (not childhood-specific) obesity in the UK media were also analysed which had been collected and described fully elsewhere[21] to enable comparison of newspaper representations of obesity in children with obesity in adults, and obesity coverage more generally. This direct comparison was enabled by the intentional similarity of the methods of data collection, coding and analysis in the two studies.

Results

Sample characteristics

Table 1 summarises the political alignment and market of each publication in the sample, in addition to the frequency of articles and front-page articles within those publications, and the variation in word count within those articles. A total of 757 articles relevant to childhood obesity were identified within the selected six publications (five of which were combined with their corresponding Sunday counterparts). The frequency of coverage of childhood obesity varied between publications, ranging from the *Independent*& *Independent on Sunday* publishing 61 relevant articles, none of which were on front pages, to the *Mirror* & *Sunday Mirror*, which published 198 relevant articles, including two front-page articles. The Daily Telegraph & Sunday Telegraph afforded the issue the greatest prominence, featuring it on their front pages nine times.

[Insert Table 1. Summary of article characteristics]

The changing frequency of relevant articles within the sample between 1996 and 2014 are illustrated in Figure 1, both overall and within each political alignment. The total number of relevant articles

For peer review only - http://bmjopen.bmj.com/site/about/guidelines.xhtml

per year rose steadily from 2 in 1996 to a high of 82 in 2008, before declining to 40 in 2010, and finally rising again to 69 articles in 2014. The peak from 2006-08 was contemporaneous with the publication of the UK Government's Foresight project report on reducing obesity[8]and its corresponding mid-term and one-year reviews.

[Insert Figure 1. Frequency of articles by year]

Definitions of the problem of childhood obesity

Table 2 illustrates the frequencies of articles mentioning specific problem definitions, drivers and solutions related to childhood obesity, and Table 3 illustrates the extent to which publications' political alignment predicted mentions of specific definitions. More than half of articles quantified childhood obesity prevalence within the UK (n=413 54.6%), and a similar proportion described obesity prevalence as rising, or having risen (n=389, 51.4%). Centre-right-aligned publications mentioned increasing prevalence significantly less frequently than centre-left publications (OR:0.59; p=0.001). Eighty (10.6%) articles quantified the prevalence of obesity outside of the UK. Approximately half of articles specifically described obesity as a health risk (n=397, 52.4%), and 102 (13.5%) described it as a burden to the National Health Service, and each of these themes were more frequent in centre-left publications (OR:0.35, p=0.010; OR:0.50, p=0.008).Childhood obesity was characterised as an economic burden to society in 74 (9.8%) articles, and significantly more so in centre-left publications (OR:0.35, p=0.010).

For peer review only

[Insert Table 2. Frequency of mentions of problem definitions, drivers, and categories of solutions]

[Insert Table 3. Likelihood of centre-right-aligned publications mentioning definitions of obesity]

Few articles (n=23, 3.0%) characterised obesity as a cosmetic problem. Twice as many articles mentioned childhood obesity in relation to women and/or girls (n=112, 14.8%) as men and/or boys (n=56, 7.4%), and men and/or boys were more likely to be mentioned in centre-left publications than centre-right publications, after adjusting for market (OR:0.43, p=0.020).

For peer review only - http://bmjopen.bmj.com/site/about/guidelines.xhtml

Presentations of potential drivers of, and solutions to, childhood obesity

Mentions of specific drivers of childhood obesity were coded and categorised as either individual (n=453, 59.8%), societal (n=214, 28.3%) or biological/genetic (n=70, 9.2%) drivers (Table 2). Societal drivers were mentioned more frequently in centre-left publications (OR:0.69, p=0.046).Frequently-mentioned individual drivers included parenting (n=246, 32.5%), diet (n=235, 31.0%) and insufficient exercise (n=224, 29.6%), while societal drivers included an abundance of unhealthy food (n=129, 17.0%), marketing (n=90, 11.9%) and insufficient health services or facilities (n=53, 7.0%).

In addition to drivers, mentions of potential solutions to childhood obesity were coded into three corresponding categories: individual (n=276, 36.5%), societal (n=214, 28.3%) and biological (n=52, 6.9%) (Table 2). Table 4 illustrates the extent to which publications' political alignment predicted mentions of specific drivers and solutions. After adjusting for publication market, centre-left publications were more likely to mention societal drivers (OR:0.69, p=0.046) and societal solutions (OR:0.54, p=0.000). Regarding specific societal drivers, centre-left publications were more likely to mention marketing (OR:0.55, p=0.030) the an abundance of fast food (OR:0.61, p=0.011), but the latter was only significant before adjusting for publication market.

[Insert Table 4. Likelihood of centre-right-aligned publications mentioning categories of driver and solution]

Time trends in presentation of drivers and solutions

Time trends in mentioning each category were analysed. Mentions of individual drivers (coefficient -0.068, p<0.001), individual solutions (coefficient -0.037, p=0.041), societal drivers (coefficient -0.097, p<0.001) and societal solutions (coefficient -0.044, p=0.012) each decreased significantly between 1996 and 2014. Neither biological/genetic drivers (coefficient -0.014, p=0.637) nor biological solutions (coefficient -0.020, p=0.558) varied significantly across the sample period.

For peer review only - http://bmjopen.bmj.com/site/about/guidelines.xhtml

For peer review only

Figure 2 illustrates the trends in individual and societal drivers and solutions. Individual drivers were mentioned particularly frequently (82-100%) between 1998 and 2000, before declining to between 46% and 67% of articles between 2004 and 2014. Mentions of individual solutions peaked at 83% in 2000, and subsequently declined, comprising 25-38% articles between 2007 and 2014. Mentions of societal drivers peaked at 67% in 2000, followed by a lower peak of 61% in 2002 and a subsequent lengthy decline to a low of 8% in 2012. Mentions of societal solutions exhibited a less linear decline than other categories, with peaks in 1998 (73%), 2004 (71%) and 2008 (66%), interspersed with declines. Notably, societal solutions were more commonly mentioned than individual solutions from 2007-2014.

[Insert Figure 2. Trends in individual and societal drivers and solutions]

Representations of childhood obesity in comparison to adult and general population obesity

The data collected for this study were compared with data collected in Hilton and colleagues' 2012 study of newspaper representations of obesity in the general population.[21] Supporting information Figure S1 illustrates the yearly frequency of articles in each study's main sample, as well as a subsample of the previous study's data that excludes all articles that mentioned children. This represents a means of comparing representations of childhood obesity with representations of adult and non-age-specific obesity. Figure S1 suggests that childhood obesity received less newspaper coverage than adult obesity in every year covered by the two datasets, with the exception of 1999. The longer time period represented in the current study suggests that the decrease in publication frequency in 2008-2010 observed in the previous study[21] did not continue in subsequent years, at least on the topic childhood obesity.

For peer review only

For peer review only - http://bmjopen.bmj.com/site/about/guidelines.xhtml

Supporting information Figure S2 illustrates the frequency of coverage of the two categories of driver and solution, individual and societal, within the present and prior sample.[21] Comparison of the data indicates that coverage of childhood obesity was characterised by greater focus on individual drivers and societal solutions than coverage of adult obesity, while coverage of societal drivers and individual solutions was relatively similar.

Discussion

By systematically analysing the content of 757 articles, we arrived at several key findings related to UK national newspapers' representations of childhood obesity. Coverage of the issue grew steadily from two articles in 1996 to a high of 82 articles in 2008, after which article frequency declined to 45 in 2009, before rising to a second peak of 69 in 2014. Childhood obesity was predominantly characterised as driven by individual-level factors, particularly parenting, dietary behaviours and inactivity, though societal drivers such as marketing were also identified. Similarly, there was greater focus on individual-level solutions than societal-level solutions. Societal constructions of the drivers of, and solutions to, obesity, were significantly more frequent within centre-left publications than centre-right. Analysis of time trends provided evidence of a small shift towards societal conceptualisations, with mentions of social solutions outnumbering individual solutions throughout the latter half of the sample period. Childhood obesity was frequently defined as a health risk in approximately half of articles, and was associated with females substantially more frequently than males, but more nuanced coding of gender-representation in these articles is required.

Centre-left publications' greater focus on societal constructions of the causes of, and solutions to childhood obesity, and on the societal and health service burdens of childhood obesity, are in line with the communal and individual framings associated with left- and right-wing political ideologies. Entman describes the core process of building frames as *"[selecting] some aspects of a perceived*

For peer review only

For peer review only - http://bmjopen.bmj.com/site/about/guidelines.xhtml

reality and [making] them more salient"[16], and this process is evident in UK newspaper representations of childhood obesity, with centre-left publications building frames that incorporate societal aspects of the childhood obesity problem, while centre-right publications omit them.

This research comprised a systematic analysis of a large sample of nineteen years of UK national newspaper coverage, facilitating statistical understandings of media frames of childhood obesity, including definitions, drivers and solutions. However, the research is subject to some limitations. The *Nexis* database does not archive articles from the Daily Telegraph and Sunday Telegraph prior to October 2000 and November 2000, respectively. However, the low frequency of reporting on childhood obesity prior to 2000 in the other sources in the sample during those years suggests that the absence of those two sources is unlikely to have had a relevant impact on our analysis. The method allowed quantitative analysis of a media frames across a large sample, but not the nuanced analysis of specific aspects of framing that qualitative analysis would permit.

The coding frame was extensive, but subject to certain limitations. Coding did not record the types of issues discussed by each articles, which may have been valuable given the variety of different perspectives from which the issue may be viewed. Further, while mentions of males and females in relation to obesity in children were coded, coding did not differentiate between mentions of boys with obesity, girls with obesity, male parents and female parents. Given the frequently gendered nature of societal discourse about obesity, future research may benefit from analysing gendered representations of both children and parents within news coverage of childhood obesity. Additionally, future research may benefit from widening the search scope from childhood obesity to also cover childhood overweight. Our search terms were used to replicate those in a previous study (ref to Swedish paper) as therefore do not include the term 'childhood', which could lead to some relevant articles being missed. However, test searches suggest that incorporating the term 'childhood' into the search string returns negligible additional articles from UK national newspapers, so it is unlikely that those absent articles would have substantially affected the analysis. Further

For peer review only - http://bmjopen.bmj.com/site/about/guidelines.xhtml

limitations of the research stem from decisions made about the type of content analysed. The sole focus on article text was at the cost of analysing images, which have been found to be an important aspect of media representations of obesity.[23 31 54 55] Further, the focus on newspaper content was at the expense of data from other news sources, such as television and online news, or alternative sources, such as reader comments or social media posts. We argue that our focus on the evolution of the debate over time is not well suited to the rapidly-changing online news environment, but acknowledge that incorporating other types of source could be valuable, as representations of childhood obesity have been found to vary by medium in the US.[34] Finally, while links between media representations and public perceptions are well established, content analysis can only describe content, not determine how that content is received by audiences.

This research built upon prior research examining media framing of general obesity[21] by extending the time period covered, taking a sole focus on childhood obesity, and comparing coverage of childhood obesity to that of obesity in general. As would be expected, the growth in coverage of childhood obesity from 1996 to 2008 identified in our prior research[21] was replicated in the present research, but it was found that the rise did not continue beyond 2008, although it remained at an elevated level of coverage relative to pre-2002. Further research might investigate whether the increase in article frequency in the final year of the study period is indicative of a prolonged rise in coverage beyond 2014. Although it is likely that coverage of childhood obesity in 2007-8 was elevated due to dissemination of, and activities related to, the UK Government's Foresight report *Reducing obesity: future choices*, published in October 2007,[8] this trend mirrors that found in Barry and colleagues'[34] content analysis of US television and print news coverage of childhood obesity suggesting that, despite locally-relevant policy events, trends in coverage of childhood obesity may follow transnational patterns. Barry and colleagues[34] suggest that the decline in coverage may be an example of Downs'[56] "issue attention cycle", in which public attention to a specific issue will inevitably decline regardless of whether that issue reaches any conclusion. However, one area where our findings depart from those of Barry and colleagues[34] is in individual and structural causes

For peer review only - http://bmjopen.bmj.com/site/about/guidelines.xhtml

of childhood obesity, which they found to be equally frequent within the newspaper articles in the their sample.

Both the original study by Hilton and colleagues and the present study present some evidence of a shift away from a focus on individual constructions of drivers and solutions across their respective time periods. However, comparison of the two pieces of research suggests that, in comparison to general obesity, media frames of childhood obesity have a greater tendency to attribute responsibility to individuals. The disproportionate individual-level framing of childhood obesity might be explained by the presence of parents as mediators between children and public policy. While children are vulnerable to societal and environmental pressures, and are often publicly viewed as deserving of legislative protection,[57-59] public discourse around childhood obesity may attribute greater individual responsibility to parents.[60] Hawkins and Linvill found that US news frequently identifies parents as both responsible for, and responsible for addressing, children's obesity, and conclude that this framing represents an obstacle to stimulating demand for a public policy response to the problem.[35] Boero's qualitative analysis of US media representations of childhood obesity identifies parents, and particularly mothers, as being 'under fire' for failing to foster healthy behaviours in their children.[28] Unlike in debates around unhealthy phenomena such as exposure to second-hand smoke, in which an adult lifestyle product may be perceived as unfairly invading children's spaces, feeding children occupies a complex position of being nurturing and essential, while also being a potential source of long-term health harms.[60]

For media content to drive public appetite for policy solutions to childhood obesity, media must both raise perceptions of the issue, through heightened coverage, and frame the issue as one demanding societal-level, rather than solely individual-level, solutions. Our research demonstrates that, while the salience of childhood obesity in UK national newspapers rose steadily from 1996-2008, that level of attention was not maintained subsequent to 2008, although there is reason to suggest that this may change in 2017/18 with media coverage of the incoming levy on sugar-

For peer review only - http://bmjopen.bmj.com/site/about/guidelines.xhtml

sweetened beverages in the UK.[61] While this faltering frequency of reporting may be undesirable for raising public consciousness, our analysis suggests that the frames constructed within those later years were characterised by a predominance of social solutions over individual solutions, which, if internalised by audiences, may stimulate public appetites for engaging the problem at the public policy level. Notably, this shift from individual to social framing occurred despite the well-documented complications caused by parents' roles as mediators between public policy and children's health behaviours. Taking these key findings into account, this study supports a mixed view of UK media framing of childhood obesity, in which positive changes in framing may be undermined by a decrease in salience. Those advocating for public policy responses to childhood obesity may seek to raise the issue's media profile, while continuing to promote social framings.

For peer review only

Author Statement

SH, CP and AN: study planning and conceptualisation; AN and CP: data coding; AN and CP: data analysis; AN and CP: drafting manuscript; SH: critical review of the manuscript.

Data Statement

Data were accessed from the Nexis newspaper database at https://www.nexis.com

For peer review only - http://bmjopen.bmj.com/site/about/guidelines.xhtml

TABLES:

Table 1. Summary of article characteristics

Publication	Political alignment	Market	All articles		Front-page articles		Word count		
			n	%*	n	%**	1st quartile	Median	3rd quartile
Guardian & Observer	Centre-left	Quality	109	14.4	5	4.6	457	680	907
Independent & Independent on Sunday	Centre-left	Quality	61	8.1	0	-	247	474	690
Mirror & Sunday Mirror	Centre-left	Tabloid	198	26.2	2	1.0	121	219	459
Daily Telegraph & Sunday Telegraph	Centre-right	Quality	107	14.1	9	8.4	182	346	502
Daily Mail & Mail on Sunday	Centre-right	Middle-market	134	17.7	6	4.5	263	438	672
Sun	Centre-right	Tabloid	148	19.6	0	-	98	195	337
		**Total**	**757**	**100.0**	**22**	**2.9**	**151**	**325**	**595**

*percentage within whole sample
**percentage of front-page articles within publication

For peer review only

Table 2. Frequency of mentions of problem definitions, drivers, and categories of solutions

Theme	Total (n=757) n	%	Inter-rater agreement*
**Problem definitions**			
Quantifies obesity prevalence within the UK	413	54.6	0.834
Quantifies obesity prevalence elsewhere	80	10.6	0.814
Mentions increase in obesity rates	389	51.4	0.940
Mentions obesity as a risk to health	397	52.4	0.893
Mentions obesity as a cosmetic problem	23	3.0	0.850
Mentions obesity as a burden to NHS	102	13.5	0.814
Mentions obesity as an economic burden to society	32	4.2	0.630
Mentions socio-economic and geographical differences	74	9.8	0.706
Mentions women and/or girls	112	14.8	0.706
Mentions men and/or boys	56	7.4	0.706
Obesity is not a problem, over-hyped etc.	93	12.3	0.850
Mentions discrimination, bullying or stigmatisation	70	9.2	1.000
**Drivers of obesity**			
Overall drivers			
Any drivers mentioned	522	69.0	n/a**
Any biological/genetic driver mentioned	70	9.2	n/a**
Any individual driver mentioned	453	59.8	n/a**
Any societal driver mentioned	214	28.3	n/a**
Individual drivers			
Mentions poor diet, overeating	235	31.0	0.857
Mentions poor self-control, willpower or choices	60	7.9	0.680
Mentions insufficient exercise, sedentary lifestyle	224	29.6	0.919
Mentions parenting shortcomings	246	32.5	0.939
Societal drivers			
Mentions an abundance of processed/fast food	129	17.0	0.752
Mentions a lack of health services or facilities	53	7.0	0.945
Mentions food/drink advertising and promotions	90	11.9	1.000
**Solutions to obesity**			
Any solution mentioned	538	71.1	n/a**
Individual solution mentioned	276	36.5	0.920
Societal solution mentioned	214	28.3	0.839
Biological solution mentioned	52	6.9	1.000

*Cohen's kappa test of inter-rater agreement.
**Agreement was not calculated for these variables as they were computed from other, manually-coded variables

For peer review only - http://bmjopen.bmj.com/site/about/guidelines.xhtml

Table 3. Likelihood of centre-right-aligned publications mentioning definitions of obesity

	Unadjusted			Adjusted*		
	OR	95% CI	P-value	OR	95% CI	P-value
**Problem definitions**						
Quantifies obesity prevalence within the UK	0.97	0.73-1.30	0.858	0.92	0.67-1.27	0.608
Quantifies obesity prevalence elsewhere	0.63	0.40-1.01	0.057	0.59	0.34-1.03	0.065
Mentions increase in obesity rates	0.70	0.52-0.93	**0.014**	0.59	0.42-0.81	**0.001**
Mentions obesity as a risk to health	1.02	0.77-1.36	0.885	0.88	0.64-1.22	0.456
Mentions obesity as a cosmetic problem	0.40	0.16-0.99	**0.048**	0.35	0.11-1.05	0.061
Mentions obesity as a burden to NHS	0.57	0.37-0.87	**0.009**	0.50	0.30-0.83	**0.008**
Mentions obesity as an economic burden to society	0.36	0.19-0.70	**0.003**	0.35	0.16-0.78	**0.010**
Mentions socio-economic and geographical differences	0.62	0.38-1.00	0.051	0.85	0.51-1.43	0.547
Mentions women and/or girls	0.86	0.58-1.29	0.467	0.77	0.48-1.23	0.271
Mentions men and/or boys	0.59	0.34-1.03	0.062	0.43	0.22-0.88	**0.020**
Obesity is not a problem, over-hyped etc.	0.75	0.29-1.93	0.552	0.73	0.25-2.15	0.565
Mentions discrimination, bullying or stigmatisation	0.56	0.36-0.87	**0.010**	0.44	0.25-0.76	**0.003**

*Adjusted for publication market

For peer review only - http://bmjopen.bmj.com/site/about/guidelines.xhtml

Table 4. Likelihood of centre-right-aligned publications mentioning categories of driver and solution

	Unadjusted			Adjusted*		
	OR	95% CI	P-value	OR	95% CI	P-value
**Drivers of obesity**						
Overall drivers						
Any drivers mentioned						
	0.90	0.66-1.23	0.505	0.78	0.56-1.10	0.162
Any biological/genetic driver mentioned						
	0.73	0.45-1.20	0.214	0.85	0.49-1.46	0.557
Any individual driver mentioned						
	1.00	0.75-1.34	0.974	0.84	0.61-1.16	0.292
Any societal driver mentioned						
	0.62	0.45-0.86	**0.004**	0.69	0.48-0.99	**0.046**
Individual drivers						
Mentions poor diet, overeating						
	0.73	0.54-0.99	**0.045**	0.65	0.46-0.93	**0.018**
Mentions poor self-control, willpower or choices						
	0.61	0.35-1.04	0.068	0.71	0.39-1.28	0.255
Mentions insufficient exercise, sedentary lifestyle						
	0.75	0.55-1.03	0.077	0.67	0.47-0.97	**0.032**
Mentions parenting shortcomings						
	1.14	0.84-1.55	0.386	1.08	0.77-1.52	0.660
Societal drivers						
Mentions an abundance of processed/fast food						
	0.61	0.41-0.89	**0.011**	0.73	0.48-1.12	0.153
Mentions a lack of health services or facilities						
	0.90	0.52-1.58	0.725	0.87	0.46-1.65	0.671
Mentions food/drink advertising and promotions						
	0.56	0.36-0.88	**0.012**	0.55	0.32-0.94	**0.030**
**Solutions to obesity**						
Biological						
	0.73	0.42-1.29	0.286	0.54	0.26-1.09	0.087
Individual						
	0.90	0.67-1.20	0.464	0.90	0.64-1.25	0.527
Societal						
	0.62	0.46-0.83	**0.001**	0.54	0.39-0.75	**0.000**

*Adjusted for publication market

For peer review only

For peer review only - http://bmjopen.bmj.com/site/about/guidelines.xhtml

For peer review only

For peer review only - http://bmjopen.bmj.com/site/about/guidelines.xhtml

References

1. Ebbeling CB, Pawlak DB, Ludwig DS. Childhood obesity: public-health crisis, common sense cure. Lancet 2002;**360**(9331):473-82
2. Ng M, Fleming T, Robinson M, et al. Global, regional, and national prevalence of overweight and obesity in children and adults during 1980–2013: a systematic analysis for the Global Burden of Disease Study 2013. Lancet 2014;**384**(9945):766-81
3. NHS Digital. Statistics on Obesity, Physical Activity and Diet, 2017.
4. Scottish Government. The Scottish Health Survey: 2015 edition. Edinburgh: Scottish Government, 2015.
5. Simmonds M, Llewellyn A, Owen CG, et al. Predicting adult obesity from childhood obesity: a systematic review and meta-analysis. Obesity Reviews 2016;**17**(2):95-107 doi: 10.1111/obr.12334[published Online First: Epub Date]|.
6. HM Government. Childhood obesity: a plan for action, 2017.
7. Scottish Government. Increase the proportion of healthy weight children. Secondary Increase the proportion of healthy weight children 2016. http://www.gov.scot/About/Performance/scotPerforms/indicator/healthyweight.
8. Butland B, Jebb S, Kopelman P, et al. Foresight. Tackling Obesities: Future Choices - Project report. 2nd ed. London: Government Office for Science, 2007.
9. Friedman DA. Public health regulation and the limits of paternalism. Conn L Rev 2013;**46**:1687
10. Swinburn BA, Sacks G, Hall KD, et al. The global obesity pandemic: shaped by global drivers and local environments. Lancet 2011;**378**(9793):804-14
11. Swinburn BA. Obesity prevention: the role of policies, laws and regulations. Australia and New Zealand health policy 2008;**5**(1):12
12. Hilbert A, Rief W, Braehler E. What determines public support of obesity prevention? J Epidemiol Community Health 2007;**61**(7):585
13. Evans WD, Finkelstein EA, Kamerow DB, et al. Public perceptions of childhood obesity. Am J Prev Med 2005;**28**(1):26-32 doi: http://dx.doi.org/10.1016/j.amepre.2004.09.008[published Online First: Epub Date]|.
14. McCombs ME, Shaw DL. The agenda-setting function of mass media. Public Opin Q 1972;**36**(2):176-87
15. Scheufele DA, Tewksbury D. Framing, agenda setting, and priming: The evolution of three media effects models. J Commun 2007;**57**(1):9-20
16. Entman RM. Framing: Toward clarification of a fractured paradigm. J Commun 1993;**43**(4):51-58
17. Barry CL, Brescoll VL, Gollust SE. Framing Childhood Obesity: How Individualizing the Problem Affects Public Support for Prevention. Polit Psychol 2013:n/a-n/a doi: 10.1111/pops.12018[published Online First: Epub Date]|.
18. Gollust SE, Niederdeppe J, Barry CL. Framing the Consequences of Childhood Obesity to Increase Public Support for Obesity Prevention Policy. Am J Public Health 2013;**103**(11):e96-e102 doi: 10.2105/ajph.2013.301271[published Online First: Epub Date]|.
19. Barry CL, Brescoll VL, Brownell KD, et al. Obesity Metaphors: How Beliefs about the Causes of Obesity Affect Support for Public Policy. Milbank Q 2009;**87**(1):7-47 doi: 10.1111/j.1468-0009.2009.00546.x[published Online First: Epub Date]|.
20. Boyce T. The media and obesity. Obesity Reviews 2007;**8**:201-05 doi: 10.1111/j.1467-789X.2007.00342.x[published Online First: Epub Date]|.
21. Hilton S, Patterson C, Teyhan A. Escalating coverage of obesity in UK newspapers: the evolution and framing of the “obesity epidemic” from 1996 to 2010. Obes 2012;**20**(8):1688-95
22. Hilbert A, Ried J. Obesity in Print: An Analysis of Daily Newspapers. Obesity Facts 2009;**2**(1):46-51
23. Patterson C, Hilton S. Normalisation and Stigmatisation of Obesity in UK Newspapers: a Visual Content Analysis. The Open Obesity Journal 2013;**5**:82-91 doi: 10.2174/1876823720131001011[published Online First: Epub Date]|.

For peer review only - http://bmjopen.bmj.com/site/about/guidelines.xhtml

24. Flint SW, Hudson J, Lavallee D. The portrayal of obesity in UK national newspapers. Stigma and Health 2016;**1**(1):16
25. Islam S, Fitzgerald L. Indigenous obesity in the news: a media analysis of news representation of obesity in Australia's Indigenous population. BMC obesity 2016;**3**(1):30
26. Atanasova D. Obesity frames in British and German online newspapers, 2009-2011. Department of Media and Communication, 2015.
27. Bonfiglioli C. Overweight and Obesity in TV News. Values and Choices in Television Discourse: Springer, 2015:87-108.
28. Boero N. All the news that's fat to print: the American "obesity epidemic" and the media. Qualitative Sociology 2007;**30**(1):41-60
29. Lawrence RG. Framing obesity: The evolution of news discourse on a public health issue. Harvard International Journal of Press/Politics 2004;**9**(3):56-75 doi: citeulike-article-id:4228152[published Online First: Epub Date]|.
30. Kim S-H, Willis LA. Talking about Obesity: News Framing of Who Is Responsible for Causing and Fixing the Problem. Journal of Health Communication 2007;**12**(4):359-76 doi: 10.1080/10810730701326051[published Online First: Epub Date]|.
31. Gollust SE, Eboh I, Barry CL. Picturing obesity: Analyzing the social epidemiology of obesity conveyed through US news media images. Soc Sci Med 2012;**74**(10):1544-51
32. Jeong M, Gilmore JS, Bleakley A, et al. Local news media framing of obesity in the context of a sugar-sweetened beverage reduction media campaign. Journal of nutrition education and behavior 2014;**46**(6):583-88
33. Varava K. Visual framing, stigmatization, race, and obesity: Examining television news presentation of stories about obesity and stories about health. Communication Studies 2016;**67**(5):509-29
34. Barry CL, Jarlenski M, Grob R, et al. News media framing of childhood obesity in the United States from 2000 to 2009. Pediatrics 2011;**128**(1):132-45 doi: peds.2010-3924 [pii]

10.1542/peds.2010-3924[published Online First: Epub Date]|.
35. Hawkins KW, Linvill DL. Public health framing of news regarding childhood obesity in the United States. Health communication 2010;**25**(8):709-17
36. Bastian A. Representations of childhood obesity in Australian newsprint media and academic literature. Aust N Z J Public Health 2011;**35**(2):135-39
37. Maher J, Fraser S, Wright J. Framing the mother: childhood obesity, maternal responsibility and care. J Gend Stud 2010;**19**(3):233-47
38. Kalin SR, Fung TT. Comparison of child obesity prevention and control content in mainstream and Spanish-language US parenting magazines. Journal of the Academy of Nutrition and Dietetics 2013;**113**(1):133-40
39. Boero N. Obesity in the media: Social science weighs in. Critical Public Health 2013;**23**(3):371-80
40. Douglas F, Clark J, Craig L, et al. "It'sa balance of just getting things right": mothers' views about pre-school childhood obesity and obesity prevention in Scotland. BMC public health 2014;**14**(1):1009
41. Harris JK, Moreland-Russell S, Tabak RG, et al. Communication About Childhood Obesity on Twitter. Am J Public Health 2014;**104**(7):e62-e69 doi: 10.2105/ajph.2013.301860[published Online First: Epub Date]|.
42. So J, Prestin A, Lee L, et al. What Do People Like to "Share" About Obesity? A Content Analysis of Frequent Retweets About Obesity on Twitter. Health Communication 2016;**31**(2):193-206 doi: 10.1080/10410236.2014.940675[published Online First: Epub Date]|.
43. Tobitt C. National newspaper ABCs: Industry-wide circulation decline continues as Metro and Sun top the table. Secondary National newspaper ABCs: Industry-wide circulation decline continues as Metro and Sun top the table 2018. https://www.pressgazette.co.uk/national-newspaper-abcs-industry-wide-circulation-decline-continues-as-metro-and-sun-top-the-table/.

For peer review only - http://bmjopen.bmj.com/site/about/guidelines.xhtml

44. Tobitt C. The Sun overtakes Mail Online to become UK's biggest online newsbrand, latest Comscore data shows. Secondary The Sun overtakes Mail Online to become UK's biggest online newsbrand, latest Comscore data shows 2018. https://www.pressgazette.co.uk/the-sun-overtakes-mail-online-to-become-uks-biggest-online-newspaper-brand-latest-comscore-data-shows/.
45. McCombs M. *Setting the agenda: Mass media and public opinion*: John Wiley & Sons, 2018.
46. Harder RA, Sevenans J, Van Aelst P. Intermedia agenda setting in the social media age: How traditional players dominate the news agenda in election times. The International Journal of Press/Politics 2017;**22**(3):275-93
47. Press Gazette. National newspaper print ABCs for Jan 2017: Times and Observer both boost print sales year on year. Secondary National newspaper print ABCs for Jan 2017: Times and Observer both boost print sales year on year 2017. http://www.pressgazette.co.uk/national-newspaper-print-abcs-for-jan-2017-observer-up-year-on-year-the-sun-is-fastest-riser-month-on-month/.
48. Ponsford D. Daily Mail and Sun launch front-page attacks on Corbyn as Fleet Street lines up behind Theresa May. Secondary Daily Mail and Sun launch front-page attacks on Corbyn as Fleet Street lines up behind Theresa May 2017. https://www.pressgazette.co.uk/daily-mail-and-sun-launch-front-page-attacks-on-corbyn-as-fleet-street-lines-up-behind-theresa-may/.
49. Smith M. How left or right-wing are the UK's newspapers? Secondary How left or right-wing are the UK's newspapers? 2017. https://yougov.co.uk/news/2017/03/07/how-left-or-right-wing-are-uks-newspapers/.
50. Kellner P. General election 2015: how Britain really voted. Secondary General election 2015: how Britain really voted 2015. https://yougov.co.uk/topics/politics/articles-reports/2015/06/08/general-election-2015-how-britain-really-voted.
51. Landis JR, Koch GG. The measurement of observer agreement for categorical data Biometrics 1977;**33**(1):159-74
52. Patterson C, Semple S, Wood K, et al. A quantitative content analysis of UK newsprint coverage of proposed legislation to prohibit smoking in private vehicles carrying children. BMC Public Health 2015;**15**:760
53. Patterson C, Katikireddi SV, Wood K, et al. Representations of minimum unit pricing for alcohol in UK newspapers: a case study of a public health policy debate. J Public Health 2014;**37**(1):40-49
54. Heuer CA, McClure KJ, Puhl RM. Obesity stigma in online news: A visual content analysis. Journal of Health Communication 2011;**16**(9):976-87
55. McClure KJ, Puhl RM, Heuer CA. Obesity in the news: Do photographic images of obese persons influence antifat attitudes? Journal of health communication 2011;**16**(4):359-71
56. Downs A. Up and down with ecology: the "issue-attention cycle". The public 1972
57. Schneider A, Ingram H. Social construction of target populations: Implications for politics and policy. Am Polit Sci Rev 1993:334-47
58. Freeman B, Chapman S, Storey P. Banning smoking in cars carrying children: an analytical history of a public health advocacy campaign. Aust N Z J Public Health 2008;**32**(1):60-65
59. Hilton S, Wood K, Bain J, et al. Newsprint coverage of smoking in cars carrying children: a case study of public and scientific opinion driving the policy debate. BMC Public Health 2014;**14**(1):1116
60. Schwartz MB, Puhl R. Childhood obesity: a societal problem to solve. Obesity reviews 2003;**4**(1):57-71
61. Buckton CH, Patterson C, Hyseni L, et al. The palatability of sugar-sweetened beverage taxation: A content analysis of newspaper coverage of the UK sugar debate. PLOS ONE 2018;**13**(12):e0207576 doi: 10.1371/journal.pone.0207576[published Online First: Epub Date]|.

For peer review only - http://bmjopen.bmj.com/site/about/guidelines.xhtml

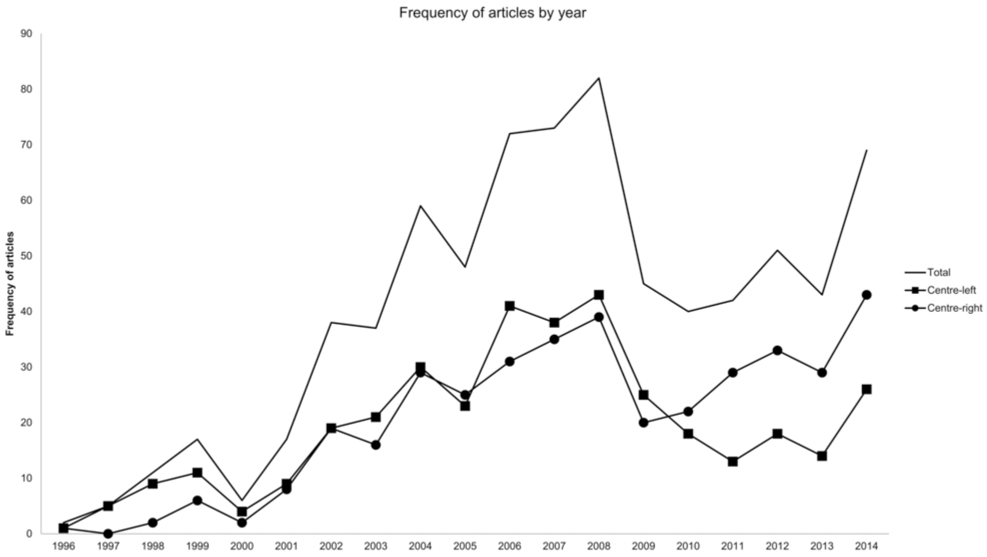

Figure 1. Frequency of articles by year

84x47mm (300 x 300 DPI)

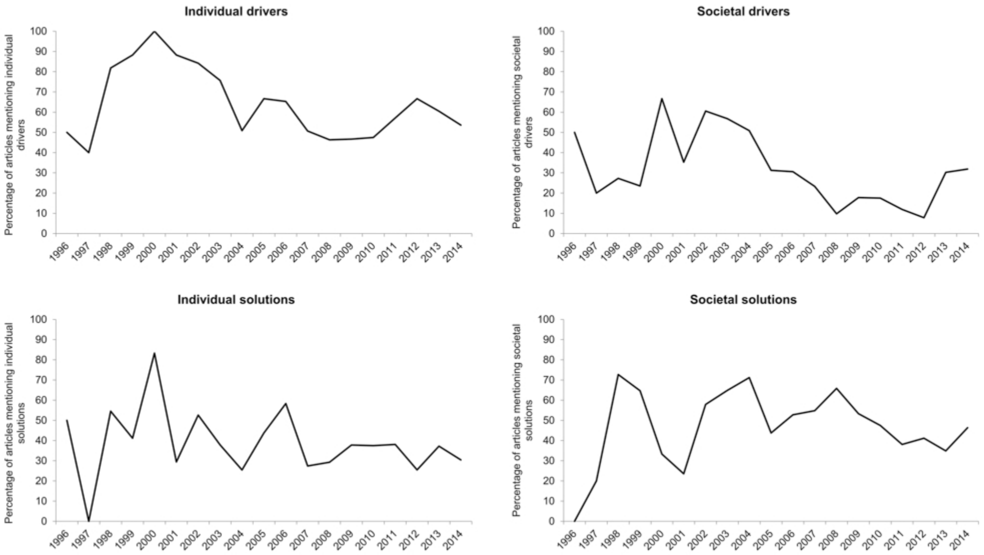

Figure 2. Trends in individual and societal drivers and solutions

84x47mm (300 x 300 DPI)

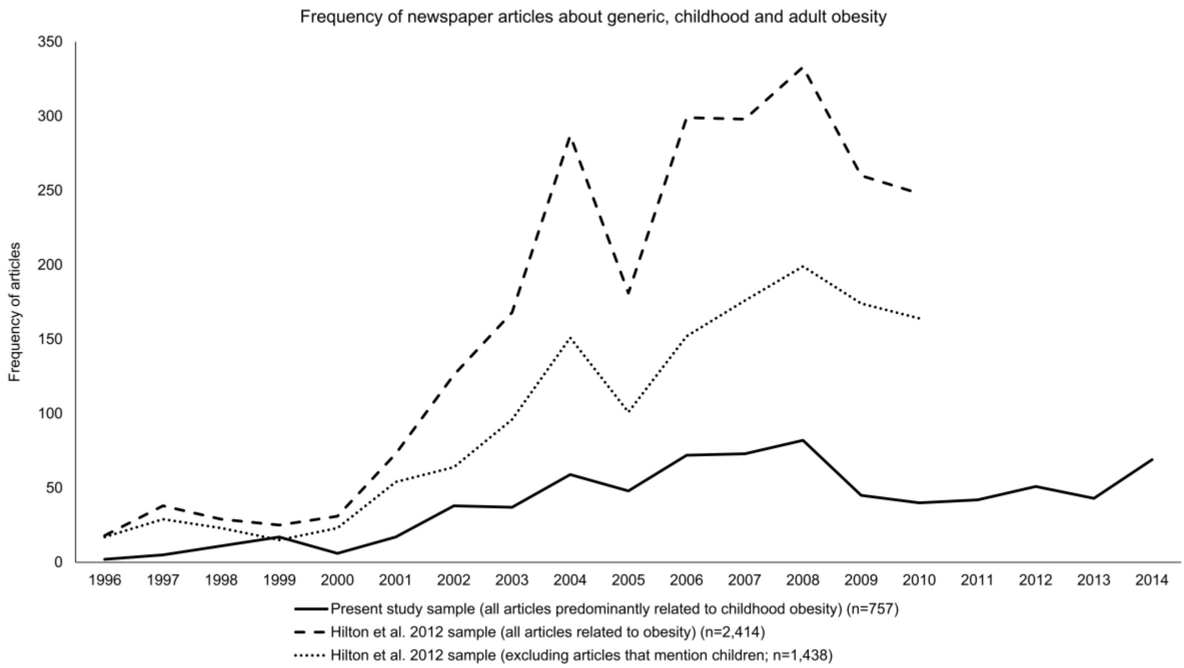
Frequency of newspaper articles about generic, childhood and adult obesity
Frequency of articles
0
50
100
150
200
250
300
350
1996 1997 1998 1999 2000 2001 2002 2003 2004 2005 2006 2007 2008 2009 2010 2011 2012 2013 2014
Present study sample (all articles predominantly related to childhood obesity) (n=757)
Hilton et al. 2012 sample (all articles related to obesity) (n=2,414)
Hilton et al. 2012 sample (excluding articles that mention children; n=1,438)

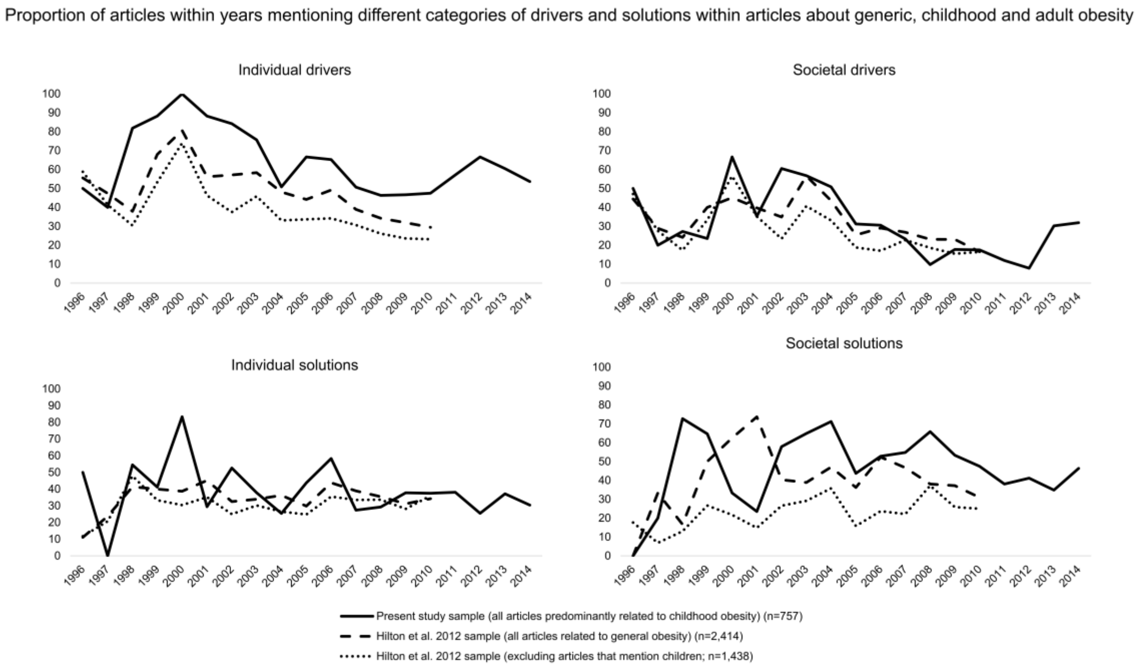
Proportion of articles within years mentioning different categories of drivers and solutions within articles about generic, childhood and adult obesity
Individual drivers
Societal drivers
Individual solutions
Societal solutions
0
10
20
30
40
50
60
70
80
90
100
1996 1997 1998 1999 2000 2001 2002 2003 2004 2005 2006 2007 2008 2009 2010 2011 2012 2013 2014
Present study sample (all articles predominantly related to childhood obesity) (n=757)
Hilton et al. 2012 sample (all articles related to general obesity) (n=2,414)
Hilton et al. 2012 sample (excluding articles that mention children; n=1,438)

No	Item	Guide and description	Reported on page
**1**	Aim	State the research question the synthesis addresses.	Title and 8
**2**	Synthesis methodology	Identify the synthesis methodology or theoretical framework which underpins the synthesis, and describe the rationale for choice of methodology *(e.g. meta-ethnography, thematic synthesis, critical interpretive synthesis, grounded theory synthesis, realist synthesis, meta-aggregation, meta-study, framework synthesis).*	Title and 8
**3**	Approach to searching	Indicate whether the search was pre-planned (*comprehensive search strategies to seek all available studies)* or iterative (*to seek all available concepts until they theoretical saturation is achieved).*	8,9,10
**4**	Inclusion criteria	Specify the inclusion/exclusion criteria *(e.g. in terms of population, language, year limits, type of publication, study type).*	9
**5**	Data sources	Describe the information sources used (e.g. *electronic databases (MEDLINE, EMBASE, CINAHL, psycINFO, Econlit), grey literature databases (digital thesis, policy reports), relevant organisational websites, experts, information specialists, generic web searches (Google Scholar) hand searching, reference lists)* and when the searches conducted; provide the rationale for using the data sources.	9

No	Item	Guide and description	Reported on page
**6**	Electronic Search strategy	Describe the literature search *(e.g. provide electronic search strategies with population terms, clinical or health topic terms, experiential or social phenomena related terms, filters for qualitative research, and search limits)*.	9
**7**	Study screening methods	Describe the process of study screening and sifting *(e.g. title, abstract and full text review, number of independent reviewers who screened studies)*.	9
**8**	Study characteristics	Present the characteristics of the included studies *(e.g. year of publication, country, population, number of participants, data collection, methodology, analysis, research questions)*.	9
**9**	Study selection results	Identify the number of studies screened and provide reasons for study exclusion *(e,g, for comprehensive searching, provide numbers of studies screened and reasons for exclusion indicated in a figure/flowchart; for iterative searching describe reasons for study exclusion and inclusion based on modifications t the research question and/or contribution to theory development)*.	9
**10**	Rationale for appraisal	Describe the rationale and approach used to appraise the included studies or selected findings *(e.g. assessment of conduct (validity and robustness), assessment of reporting*	10

No	Item	Guide and description	Reported on page
		(transparency), assessment of content and utility of the findings).	
**11**	Appraisal items	State the tools, frameworks and criteria used to appraise the studies or selected findings *(e.g. Existing tools: CASP, QARI, COREQ, Mays and Pope* [25]*; reviewer developed tools; describe the domains assessed: research team, study design, data analysis and interpretations, reporting).*	10
**12**	Appraisal process	Indicate whether the appraisal was conducted independently by more than one reviewer and if consensus was required.	10
**13**	Appraisal results	Present results of the quality assessment and indicate which articles, if any, were weighted/excluded based on the assessment and give the rationale.	NA
**14**	Data extraction	Indicate which sections of the primary studies were analysed and how were the data extracted from the primary studies? *(e.g. all text under the headings "results /conclusions" were extracted electronically and entered into a computer software).*	10
**15**	Software	State the computer software used, if any.	10
**16**	Number of reviewers	Identify who was involved in coding and analysis.	10

No	Item	Guide and description	Reported on page
**17**	Coding	Describe the process for coding of data *(e.g. line by line coding to search for concepts).*	10
**18**	Study comparison	Describe how were comparisons made within and across studies *(e.g. subsequent studies were coded into pre-existing concepts, and new concepts were created when deemed necessary).*	11
**19**	Derivation of themes	Explain whether the process of deriving the themes or constructs was inductive or deductive.	NA
**20**	Quotations	Provide quotations from the primary studies to illustrate themes/constructs, and identify whether the quotations were participant quotations of the author's interpretation.	NA
**21**	Synthesis output	Present rich, compelling and useful results that go beyond a summary of the primary studies (e.g. *new interpretation, models of evidence, conceptual models, analytical framework, development of a new theory or construct).*	15-19?

BMJ Open

Media framing of childhood obesity: a content analysis of UK newspapers from 1996-2014

Journal:	*BMJ Open*
Manuscript ID	bmjopen-2018-025646.R2
Article Type:	Research
Date Submitted by the Author:	27-Feb-2019
Complete List of Authors:	Nimegeer, Amy; Glasgow University, MRC/CSO Social and Public Health Sciences<br>Patterson, Chris; University of Glasgow, MRC/CSO Social & Public Health Sciences Unit<br>Hilton , Shona; University of Glasgow, MRC/CSO Social & Public Health Sciences Unit
**Primary Subject Heading**:	Communication
Secondary Subject Heading:	Sociology, Public health
Keywords:	Health policy < HEALTH SERVICES ADMINISTRATION & MANAGEMENT, MEDICAL JOURNALISM, Community child health < PAEDIATRICS, PAEDIATRICS, PUBLIC HEALTH, STATISTICS & RESEARCH METHODS

SCHOLARONE™
Manuscripts

TITLE: Media framing of childhood obesity: a content analysis of UK newspapers from 1996-2014

AUTHORS: Amy Nimegeer[1], Chris Patterson[1], Shona Hilton[1]

AFFILIATION: [1]MRC/CSO Social and Public Health Sciences Unit, University of Glasgow, 200 Renfield Street, Glasgow, G2 3QB, United Kingdom

KEYWORDS: Obesity, Children, Media, content analysis

RUNNING TITLE: UK Media framing of childhood obesity

CONTACT INFORMATION:

Dr Amy Nimegeer, MRC/CSO Social and Public Health Sciences Unit, University of Glasgow, 200 Renfield Street, Glasgow, G2 3QB, United Kingdom

amy.nimegeer@glasgow.ac.uk

Emails for additional authors: chris.patterson@glasgow.ac.uk ; shona.hilton@glasgow.ac.uk

WORD COUNT (excluding cover page, abstract, references, tables, and figures): 4166

For peer review only

For peer review only - http://bmjopen.bmj.com/site/about/guidelines.xhtml

Abstract

Background: Media can influence public and policymakers' perceptions of causes of, and solutions to, public health issues through selective presentation and framing. Childhood obesity is a health issue with both individual- and societal-level drivers and solutions, but public opinion and mass media representations of obesity have typically focused on individual-level framings, at the cost of acknowledgement of a need for regulatory action.

Objective and Setting: To understand the salience and framing of childhood obesity across 19 years of UK national newspaper content.

Design and Outcome Measures: Quantitative content analysis of 757 articles about childhood obesity obtained from six daily and five Sunday newspapers. Articles were coded manually for definitions, drivers and potential solutions. Data were analysed statistically, including analysis of time trends and variations by political alignment of source.

Results: The frequency of articles grew from a low of two in 1996 to a peak of 82 in 2008, before declining to 40 in 2010. Individual-level drivers (59.8%) and solutions (36.5%) were mentioned more frequently than societal-level drivers (28.3%) and solutions (28.3%) across the sample, but societal solutions were mentioned more frequently during the final eight years, coinciding with a marked decline in yearly frequency of articles.

Conclusions: Increased focus on societal solutions aligns with public health goals, but coincided with a reduction in the issue's salience in the media. Those advocating public policy solutions to childhood obesity may benefit from seeking to raise the issue's media profile while continuing to promote structural conceptualisations of childhood obesity.

For peer review only - http://bmjopen.bmj.com/site/about/guidelines.xhtml

Article Summary

Strengths and Limitations of this Study

Strengths

- Methodology includes systematic analysis of a large sample of nineteen years of UK national newspaper coverage, facilitating statistical understandings of media frames of childhood obesity, including definitions, drivers and solutions
- Features robust manual coding and links to pre-existing dataset to strengthen analysis

Weaknesses

- Quantitative media content analysis is inherently less sensitive to nuance than qualitative analysis, and our analysis excluded some aspects of media content, such as images, which may influence readers' interpretations of the text they accompany
- Content analysis is a means of documenting what messages are presented by media, but cannot tell us how these messages are received by audiences

Funding Statement

AN, CP and SH's time for this research was funded by the Informing Healthy Public Policy programme (MC_UU_12017-15 and SPHSU15) of the MRC/CSO Social and Public Health Sciences Unit, University of Glasgow. The funding bodies had no role in the design, collection, analysis or interpretation of this study.

Competing Interests

SH, CP and AN declare no conflicts of interest.

Acknowledgements

For peer review only - http://bmjopen.bmj.com/site/about/guidelines.xhtml

The authors would like to thank Dr Jonathan Olsen of the MRC/CSO Social and Public Health Sciences Unit, University of Glasgow, for his advice regarding the conduct of multiple logistic regression analysis. We would also like to acknowledge the contribution of Dr Marie Löf and Dr Mark Daku to the design of this study.

For peer review only

For peer review only - http://bmjopen.bmj.com/site/about/guidelines.xhtml

Introduction

Childhood obesity has been described as an international epidemic due to its high prevalence and rapid growth in numerous countries.[1] Globally, 13.4% of girls and 12.9% of boys in Low Income Countries, and 22.6% of girls, and 23.8% of boys in High Income Countries (classified by the World Bank) were classified as overweight or obese in 2013.[2] In England, one fifth of children in Reception year (age 4-5), and one third in Year 6 (age 10-11), were classified as overweight or obese in 2015/2016.[3] In Scotland, 28% of children aged 2-15 were classified as 'at risk of' overweight or obesity in 2015 [4]. Childhood obesity has a broad range of short- and long-term health consequences,[1] tends to predict adolescent and adult obesity,[5] and is socioeconomically patterned.[3] For these reasons childhood obesity has been identified as a health priority for the UK and its devolved governments.[6 7]

Childhood obesity is a complex problem, with a complex set of drivers and potential solutions ranging from the individual to the environmental.[8] Ebbeling and colleagues[1] identify a wide range of causes, but argue that the problem *"can be primarily attributed to adverse environmental factors"*, and identify a need for *"straightforward, if politically difficult"* solutions spanning homes, schools, the built environment, health care, marketing, media and politics. This multi-level package of solutions echoes Friedman's assertion that a 'full-court press' targeting 'every dimension of the problem' is necessary.[9] However, while academia and public health are united on the need to target the obesogenic environment, Swinburn and colleagues[10] state that *"governments have largely abdicated the responsibility for addressing obesity to individuals, the private sector, and non-governmental organisations",*[10] potentially due to anticipated or actual resistance, not just from corporations, but also electorates.[9 11] Indeed, public opinion research conducted in the US[12] and Germany[13] suggests that, while publics are in favour of tackling childhood obesity, they demonstrate less enthusiasm for regulative environmental interventions such as taxation. Hilbert characterises the German population

For peer review only - http://bmjopen.bmj.com/site/about/guidelines.xhtml

as *"ready for obesity prevention"*, but in need of education about the definition, prevalence and causes of obesity.[12]

The media represent a key influence on public perceptions of health issues and policies, setting the public agenda by granting differing levels of prominence to different topics[14] and influencing how those issues are understood by building frames (focuses of attention) that include constructions of problems, affected groups, drivers and solutions.[15 16] The influence of framing is well established in relation to obesity. Researchers have used experimental designs to demonstrate that 'individualised' representations of childhood obesity tend to lead participants to assign greater blame to individuals and exhibit less support for environmental regulation,[17] and that different representations of the consequences of childhood obesity can influence participants' attitudes towards policies.[18] Similarly, Barry and colleagues demonstrated that people's perceptions of obesity (as communicated through agreement with metaphor-based descriptions of obesity) predict their support for public policy interventions, illustrating how, for example, framing obesity as being driven by industry manipulation may lead to increased support for a 'junk-food tax'.[19]

The media are frequently accused of contributing to obesity, particularly childhood obesity, through its associations with sedentary behaviour, advertising of unhealthy commodities, promotion of unrealistic body image, and other mechanisms.[20] Many researchers have studied media representations of obesity in general,[21-33] but relatively few have focused specifically on representations of childhood obesity, and these have been primarily in the US and Australia. Barry and colleagues[34] studied US print and television news framing of childhood obesity, observing that coverage of the issue grew between 2000 and 2009, and that individual-level behavioural solutions to obesity were dominant, particularly on television. Similarly, Hawkins and Linvill[35] studied US newspaper framing of childhood obesity over three discrete time periods in 1991, 2001 and 2006, and identified a predominant focus on individual-level factors (both individual children and their parents) in representations of both causes and solutions.

For peer review only - http://bmjopen.bmj.com/site/about/guidelines.xhtml

Bastian[36] analysed representations of childhood obesity in both Australian newspapers and academic literature in 2009, identifying predominantly individual framing within the media, compared to a social-structural framing in academic literature. Bastian[36] recommends that public health professionals work to redirect media attention towards structural drivers of childhood obesity. Maher and colleagues[37] analysed constructions of maternal responsibilities within Australian media coverage of childhood obesity, concluding that the dominant framing *"individualises maternal and child relationships rather than seeing mothering as embedded in broader social and economic structures"*, serving a neo-liberal agenda by diminishing the responsibility of wider society. This is consistent with the disproportionate focus on individual-level solutions identified by others.[34-36 38] While coverage of obesity in both adults and children appears to be characterised by individual-framing, it is notable that with adult obesity that individual responsibility is assigned to the person with obesity, while in childhood obesity that responsibility is predominantly assigned to parents, particularly mothers.[39 40] This distinction may complicate direct comparison between adult and child obesity, and the culturally-ingrained nature of the concept that parents (or mothers) are solely responsible for their children's healthcare may represent a discursive obstacle to attempts to assign environmental solutions to childhood obesity.

In addition to traditional news media, researchers have analysed representations of childhood obesity in non-news media and new media. For example, Kalin and Fung's[38] analysis of Spanish-language US parenting magazines' representations of childhood obesity prevention and control echoes studies of news media representations of obesity, identifying greater focus on parental behaviour-change than system-level solutions, and limited recognition of social contextual factors. In recognition of the growing importance of user-generated social content and discussion, researchers have increasingly analysed content about childhood obesity on social media platforms.[41 42] While these new forms of media content represent an important aspect of the changing media landscape, traditional media outlets remain influential; despite the precipitous decline of UK print newspaper circulation,[43] the online presences of these hegemonic print news brands largely dominate online news readership,[44]

For peer review only - http://bmjopen.bmj.com/site/about/guidelines.xhtml

and typically define or legitimise news agendas for social media discussion.[45 46] However, it is also true that the relationship between news media and social media is interconnected and complex: social media trends are likely to influence the salience granted to issues by mainstream media outlets; social media posts frequently find themselves the object of news media reporting; and readers' comments on online traditional news articles can form part of the 'text' for subsequent readers. As an integral part of this complex new landscape, traditional media remain a relevant subject for media analysis, particularly when studying how representations evolve over timeframes predating the ascendancy of new media.

The aim of this study is to further understandings of media representations of childhood obesity in the UK context, using an approach informed by media framing theory,[15 16] analysing definitions of the problem and constructions of drivers and solutions. This is important because, while childhood obesity in the UK shares many similarities with that of other countries, the UK context differs in terms of several elements including health service structure and media environment. The analysis will have dual foci: the evolution of coverage between 1996 and 2014, and the relative salience of individual and societal constructions of the drivers of, and potential solutions to, childhood obesity. To our knowledge, this research will be the first empirical analysis of UK media framing of childhood obesity. This paper comprises the UK portion of a multi-country research project, the other parts of which will be reported in separate papers.

Methods

The media content analysis methods used were predominantly based on Hilton and colleagues' prior study[21] of UK newspaper framing of obesity in the general population, adapted for this study's focus on childhood obesity. This paper reports UK data that was part of a wider study that examined childhood obesity media coverage in two other international contexts; Sweden and the United States. Although content analysis is often viewed as an objective, descriptive approach, we subscribe to Krippendorff's position that even the quantitative analysis of text is inherently an interpretive act,

For peer review only - http://bmjopen.bmj.com/site/about/guidelines.xhtml

and researchers should therefore acknowledge the individual bias that can arise from that process, seeking to minimise that bias through research design, while also embracing how researchers' contextual understandings can enrich coding and analysis beyond the crude 'objective' counting of content.

Patient and Public Involvement

Due to the nature of this study, patients/public were not involved.

For peer review only

Sampling

A set of six daily newspapers and five Sunday newspapers with high circulation figures[47] and representing a variety of political alignments and markets (or 'genres') were chosen. Table 1 lists these publications and indicates their political alignments and the markets that they occupy. Markets were defined as tabloid (typically sensationalist and politically diverse, with predominantly working-class readerships), middle-market tabloid (centre-right content with predominantly older, middle-class readerships) and quality (serious tone with predominantly middle-class readerships), using a typology used in prior studies of UK newspaper content[21]. Political alignment was determined by cross-referencing data on: the political party endorsed by each publication at the 2017 UK general election;[48] readers' perceptions of newspapers' political alignment;[49] and the voting behaviours of each publications' readers in the 2015 UK general election. [50] A sample period of 1996 to 2014 was chosen to encompass the time period covered in prior research,[21] in addition to a further four years of coverage that was extended to align with the time period covered by the other countries in our wider study (which will be described fully in a separate publication).

Identifying relevant articles from the chosen publications involved an initial database search, followed by manual filtering of search results. The Nexis database was searched for the presence of both the term *'obesity' OR 'obese' OR 'fat'* and the term *'child' OR 'children' OR 'kid' OR kids'* within the headlines of articles published within the selected newspapers. Each chosen publication was

For peer review only - http://bmjopen.bmj.com/site/about/guidelines.xhtml

archived comprehensively within the Nexis database, with the exceptions of the Daily Telegraph and Sunday Telegraph prior to October 2000 and November 2000, respectively. As such, reporting from those publications during the first five years of the sample period was not represented. The initial search returned 1199 articles, which were subsequently subjected to manual application of exclusion criteria, including: less than 50% of article content focussing on childhood obesity (i.e. where more than half the article discussed another topic with only brief mention of childhood obesity); being a reader's letter; or being part of television guide section. Following exclusion, the final sample comprised 757 relevant articles.

Coding

Article content was coded quantitatively using a coding frame adapted from one initially developed by Hilton and colleagues.[21] The adapted coding frame was developed to record media frames of childhood obesity in terms of definitions of the problem, mentions of specific biological, individual and societal drivers, and biological, individual and societal solutions (itemised in Table 2). In addition, the coding frame recorded whether the article was published on the front page of the publication, and the length of the article in number of words. Articles were coded as relating to women/girls or men/boys if members of that gender were described as being specifically problematic in relation to childhood obesity (but not if rates for both genders were cited), or if the article profiled an individual of a specific gender. Coding was performed by AN and CP, and 10% of articles were double-coded blind to allow inter-rater agreement to be calculated. Cohen's kappa values for agreement on individual codes are listed in Table 2. The threshold for acceptable agreement was set at 0.61 (defined by Landis and Koch as 'substantial' or better agreement),[51] and three codes were removed due to insufficient agreement: dieting (such as fad diets) as a driver of childhood obesity; normalisation of obesity as a driver of childhood obesity; and technological developments as a driver of childhood obesity.

For peer review only - http://bmjopen.bmj.com/site/about/guidelines.xhtml

Analysis

Statistical analysis was performed in STATA. Statistical procedures included: basic descriptive statistics; Cohen's kappa test of inter-rater agreement; linear regression of relationships between publication year and mentions of different categories of drivers and solutions; and multiple logistic regression of relationships between political alignment and individual aspects of framing. The multiple logistic regressions were adjusted by publication market because the markets represented were not distributed evenly by political alignment (as is the case in the UK newspaper industry), and previous research has identified significant variation in health news coverage by publication market.[21 52 53]

Comparative analysis

Data from Hilton and colleagues' previous study on representations of general (not childhood-specific) obesity in the UK media were also analysed which had been collected and described fully elsewhere[21] to enable comparison of newspaper representations of obesity in children with obesity in adults, and obesity coverage more generally. This direct comparison was enabled by the intentional similarity of the methods of data collection, coding and analysis in the two studies.

Results

Sample characteristics

Table 1 summarises the political alignment and market of each publication in the sample, in addition to the frequency of articles and front-page articles within those publications, and the variation in word count within those articles. A total of 757 articles relevant to childhood obesity were identified within the selected six publications (five of which were combined with their corresponding Sunday counterparts). The frequency of coverage of childhood obesity varied between publications, ranging from the *Independent*& *Independent on Sunday* publishing 61 relevant articles, none of which were on front pages, to the *Mirror* & *Sunday Mirror*, which published 198 relevant articles, including two

For peer review only - http://bmjopen.bmj.com/site/about/guidelines.xhtml

For peer review only

front-page articles. The Daily Telegraph & Sunday Telegraph afforded the issue the greatest prominence, featuring it on their front pages nine times.

[Insert Table 1. Summary of article characteristics]

The changing frequency of relevant articles within the sample between 1996 and 2014 are illustrated in Figure 1, both overall and within each political alignment. The total number of relevant articles per year rose steadily from 2 in 1996 to a high of 82 in 2008, before declining to 40 in 2010, and finally rising again to 69 articles in 2014. The peak from 2006-08 was contemporaneous with the publication of the UK Government's Foresight project report on reducing obesity[8]and its corresponding mid-term and one-year reviews.

[Insert Figure 1. Frequency of articles by year]

Definitions of the problem of childhood obesity

Table 2 illustrates the frequencies of articles mentioning specific problem definitions, drivers and solutions related to childhood obesity, and Table 3 illustrates the extent to which publications' political alignment predicted mentions of specific definitions. More than half of articles quantified childhood obesity prevalence within the UK (n=413 54.6%), and a similar proportion described obesity prevalence as rising, or having risen (n=389, 51.4%). Centre-right-aligned publications mentioned increasing prevalence significantly less frequently than centre-left publications (OR:0.59; p=0.001). Eighty (10.6%) articles quantified the prevalence of obesity outside of the UK. Approximately half of articles specifically described obesity as a health risk (n=397, 52.4%), and 102 (13.5%) described it as a burden to the National Health Service, and each of these themes were more frequent in centre-left publications (OR:0.35, p=0.010; OR:0.50, p=0.008).Childhood obesity was characterised as an economic burden to society in 74 (9.8%) articles, and significantly more so in centre-left publications (OR:0.35, p=0.010).

[Insert Table 2. Frequency of mentions of problem definitions, drivers, and categories of solutions]

For peer review only - http://bmjopen.bmj.com/site/about/guidelines.xhtml

[Insert Table 3. Likelihood of centre-right-aligned publications mentioning definitions of obesity]

Few articles (n=23, 3.0%) characterised obesity as a cosmetic problem. Twice as many articles mentioned childhood obesity in relation to women and/or girls (n=112, 14.8%) as men and/or boys (n=56, 7.4%), and men and/or boys were more likely to be mentioned in centre-left publications than centre-right publications, after adjusting for market (OR:0.43, p=0.020).

Presentations of potential drivers of, and solutions to, childhood obesity

Mentions of specific drivers of childhood obesity were coded and categorised as either individual (n=453, 59.8%), societal (n=214, 28.3%) or biological/genetic (n=70, 9.2%) drivers (Table 2). Societal drivers were mentioned more frequently in centre-left publications (OR:0.69, p=0.046).Frequently-mentioned individual drivers included parenting (n=246, 32.5%), diet (n=235, 31.0%) and insufficient exercise (n=224, 29.6%), while societal drivers included an abundance of unhealthy food (n=129, 17.0%), marketing (n=90, 11.9%) and insufficient health services or facilities (n=53, 7.0%).

In addition to drivers, mentions of potential solutions to childhood obesity were coded into three corresponding categories: individual (n=276, 36.5%), societal (n=214, 28.3%) and biological (n=52, 6.9%) (Table 2). Table 4 illustrates the extent to which publications' political alignment predicted mentions of specific drivers and solutions. After adjusting for publication market, centre-left publications were more likely to mention societal drivers (OR:0.69, p=0.046) and societal solutions (OR:0.54, p=0.000). Regarding specific societal drivers, centre-left publications were more likely to mention marketing (OR:0.55, p=0.030) the an abundance of fast food (OR:0.61, p=0.011), but the latter was only significant before adjusting for publication market.

[Insert Table 4. Likelihood of centre-right-aligned publications mentioning categories of driver and solution]

For peer review only - http://bmjopen.bmj.com/site/about/guidelines.xhtml

Time trends in presentation of drivers and solutions

Time trends in mentioning each category were analysed. Mentions of individual drivers (coefficient -0.068, p<0.001), individual solutions (coefficient -0.037, p=0.041), societal drivers (coefficient -0.097, p<0.001) and societal solutions (coefficient -0.044, p=0.012) each decreased significantly between 1996 and 2014. Neither biological/genetic drivers (coefficient -0.014, p=0.637) nor biological solutions (coefficient -0.020, p=0.558) varied significantly across the sample period.

Figure 2 illustrates the trends in individual and societal drivers and solutions. Individual drivers were mentioned particularly frequently (82-100%) between 1998 and 2000, before declining to between 46% and 67% of articles between 2004 and 2014. Mentions of individual solutions peaked at 83% in 2000, and subsequently declined, comprising 25-38% articles between 2007 and 2014. Mentions of societal drivers peaked at 67% in 2000, followed by a lower peak of 61% in 2002 and a subsequent lengthy decline to a low of 8% in 2012. Mentions of societal solutions exhibited a less linear decline than other categories, with peaks in 1998 (73%), 2004 (71%) and 2008 (66%), interspersed with declines. Notably, societal solutions were more commonly mentioned than individual solutions from 2007-2014.

[Insert Figure 2. Trends in individual and societal drivers and solutions]

Representations of childhood obesity in comparison to adult and general population obesity

The data collected for this study were compared with data collected in Hilton and colleagues' 2012 study of newspaper representations of obesity in the general population.[21] Supporting information Figure S1 illustrates the yearly frequency of articles in each study's main sample, as well as a subsample of the previous study's data that excludes all articles that mentioned children. This represents a means of comparing representations of childhood obesity with representations of adult

For peer review only - http://bmjopen.bmj.com/site/about/guidelines.xhtml

and non-age-specific obesity. Figure S1 suggests that childhood obesity received less newspaper coverage than adult obesity in every year covered by the two datasets, with the exception of 1999. The longer time period represented in the current study suggests that the decrease in publication frequency in 2008-2010 observed in the previous study[21] did not continue in subsequent years, at least on the topic childhood obesity.

Supporting information Figure S2 illustrates the frequency of coverage of the two categories of driver and solution, individual and societal, within the present and prior sample.[21] Comparison of the data indicates that coverage of childhood obesity was characterised by greater focus on individual drivers and societal solutions than coverage of adult obesity, while coverage of societal drivers and individual solutions was relatively similar.

Discussion

By systematically analysing the content of 757 articles, we arrived at several key findings related to UK national newspapers' representations of childhood obesity. Coverage of the issue grew steadily from two articles in 1996 to a high of 82 articles in 2008, after which article frequency declined to 45 in 2009, before rising to a second peak of 69 in 2014. Childhood obesity was predominantly characterised as driven by individual-level factors, particularly parenting, dietary behaviours and inactivity, though societal drivers such as marketing were also identified. Similarly, there was greater focus on individual-level solutions than societal-level solutions. Societal constructions of the drivers of, and solutions to, obesity, were significantly more frequent within centre-left publications than centre-right. Analysis of time trends provided evidence of a small shift towards societal conceptualisations, with mentions of social solutions outnumbering individual solutions throughout the latter half of the sample period. Childhood obesity was frequently defined as a health risk in

For peer review only - http://bmjopen.bmj.com/site/about/guidelines.xhtml

For peer review only

approximately half of articles, and was associated with females substantially more frequently than males, but more nuanced coding of gender-representation in these articles is required.

Centre-left publications' greater focus on societal constructions of the causes of, and solutions to childhood obesity, and on the societal and health service burdens of childhood obesity, are in line with the communal and individual framings associated with left- and right-wing political ideologies. Entman describes the core process of building frames as *"[selecting] some aspects of a perceived reality and [making] them more salient"*[16], and this process is evident in UK newspaper representations of childhood obesity, with centre-left publications building frames that incorporate societal aspects of the childhood obesity problem, while centre-right publications omit them.

This research comprised a systematic analysis of a large sample of nineteen years of UK national newspaper coverage, facilitating statistical understandings of media frames of childhood obesity, including definitions, drivers and solutions. However, the research is subject to some limitations. The *Nexis* database does not archive articles from the Daily Telegraph and Sunday Telegraph prior to October 2000 and November 2000, respectively. However, the low frequency of reporting on childhood obesity prior to 2000 in the other sources in the sample during those years suggests that the absence of those two sources is unlikely to have had a relevant impact on our analysis. The method allowed quantitative analysis of a media frames across a large sample, but not the nuanced analysis of specific aspects of framing that qualitative analysis would permit.

The coding frame was extensive, but subject to certain limitations. Coding did not record the types of issues discussed by each articles, which may have been valuable given the variety of different perspectives from which the issue may be viewed. Further, while mentions of males and females in relation to obesity in children were coded, coding did not differentiate between mentions of boys with obesity, girls with obesity, male parents and female parents. Given the frequently gendered nature of societal discourse about obesity, future research may benefit from analysing gendered representations of both children and parents within news coverage of childhood obesity.

For peer review only - http://bmjopen.bmj.com/site/about/guidelines.xhtml

Additionally, future research may benefit from widening the search scope from childhood obesity to also cover childhood overweight. Our search terms were used to replicate those in a previous study (ref to Swedish paper) as therefore do not include the term 'childhood', which could lead to some relevant articles being missed. However, test searches suggest that incorporating the term 'childhood' into the search string returns negligible additional articles from UK national newspapers, so it is unlikely that those absent articles would have substantially affected the analysis. Further limitations of the research stem from decisions made about the type of content analysed. The sole focus on article text was at the cost of analysing images, which have been found to be an important aspect of media representations of obesity.[23 31 54 55] Further, the focus on newspaper content was at the expense of data from other news sources, such as television and online news, or alternative sources, such as reader comments or social media posts. We argue that our focus on the evolution of the debate over time is not well suited to the rapidly-changing online news environment, but acknowledge that incorporating other types of source could be valuable, as representations of childhood obesity have been found to vary by medium in the US.[34] Finally, while links between media representations and public perceptions are well established, content analysis can only describe content, not determine how that content is received by audiences.

This research built upon prior research examining media framing of general obesity[21] by extending the time period covered, taking a sole focus on childhood obesity, and comparing coverage of childhood obesity to that of obesity in general. As would be expected, the growth in coverage of childhood obesity from 1996 to 2008 identified in our prior research[21] was replicated in the present research, but it was found that the rise did not continue beyond 2008, although it remained at an elevated level of coverage relative to pre-2002. Further research might investigate whether the increase in article frequency in the final year of the study period is indicative of a prolonged rise in coverage beyond 2014. Although it is likely that coverage of childhood obesity in 2007-8 was elevated due to dissemination of, and activities related to, the UK Government's Foresight report *Reducing obesity: future choices*, published in October 2007,[8] this trend mirrors that found in Barry

For peer review only - http://bmjopen.bmj.com/site/about/guidelines.xhtml

and colleagues'[34] content analysis of US television and print news coverage of childhood obesity suggesting that, despite locally-relevant policy events, trends in coverage of childhood obesity may follow transnational patterns. Barry and colleagues[34] suggest that the decline in coverage may be an example of Downs'[56] "issue attention cycle", in which public attention to a specific issue will inevitably decline regardless of whether that issue reaches any conclusion. However, one area where our findings depart from those of Barry and colleagues[34] is in individual and structural causes of childhood obesity, which they found to be equally frequent within the newspaper articles in the their sample.

Both the original study by Hilton and colleagues and the present study present some evidence of a shift away from a focus on individual constructions of drivers and solutions across their respective time periods. However, comparison of the two pieces of research suggests that, in comparison to general obesity, media frames of childhood obesity have a greater tendency to attribute responsibility to individuals. The disproportionate individual-level framing of childhood obesity might be explained by the presence of parents as mediators between children and public policy. While children are vulnerable to societal and environmental pressures, and are often publicly viewed as deserving of legislative protection,[57-59] public discourse around childhood obesity may attribute greater individual responsibility to parents.[60] Hawkins and Linvill found that US news frequently identifies parents as both responsible for, and responsible for addressing, children's obesity, and conclude that this framing represents an obstacle to stimulating demand for a public policy response to the problem.[35] Boero's qualitative analysis of US media representations of childhood obesity identifies parents, and particularly mothers, as being 'under fire' for failing to foster healthy behaviours in their children.[28] Unlike in debates around unhealthy phenomena such as exposure to second-hand smoke, in which an adult lifestyle product may be perceived as unfairly invading children's spaces, feeding children occupies a complex position of being nurturing and essential, while also being a potential source of long-term health harms.[60]

For peer review only - http://bmjopen.bmj.com/site/about/guidelines.xhtml

For media content to drive public appetite for policy solutions to childhood obesity, media must both raise perceptions of the issue, through heightened coverage, and frame the issue as one demanding societal-level, rather than solely individual-level, solutions. Our research demonstrates that, while the salience of childhood obesity in UK national newspapers rose steadily from 1996-2008, that level of attention was not maintained subsequent to 2008, although there is reason to suggest that this may change in 2017/18 with media coverage of the incoming levy on sugar-sweetened beverages in the UK.[61] While this faltering frequency of reporting may be undesirable for raising public consciousness, our analysis suggests that the frames constructed within those later years were characterised by a predominance of social solutions over individual solutions, which, if internalised by audiences, may stimulate public appetites for engaging the problem at the public policy level. Notably, this shift from individual to social framing occurred despite the well-documented complications caused by parents' roles as mediators between public policy and children's health behaviours. Taking these key findings into account, this study supports a mixed view of UK media framing of childhood obesity, in which positive changes in framing may be undermined by a decrease in salience. Those advocating for public policy responses to childhood obesity may seek to raise the issue's media profile, while continuing to promote social framings.

Author Statement

SH, CP and AN: study planning and conceptualisation; AN and CP: data coding; AN and CP: data analysis; AN and CP: drafting manuscript; SH: critical review of the manuscript.

Data Statement

For peer review only - http://bmjopen.bmj.com/site/about/guidelines.xhtml

Data were accessed from the Nexis newspaper database at https://www.nexis.com

For peer review only

For peer review only - http://bmjopen.bmj.com/site/about/guidelines.xhtml

TABLES:

Table 1. Summary of article characteristics

Publication	Political alignment	Market	All articles n	All articles %*	Front-page articles n	Front-page articles %**	Word count 1st quartile	Word count Median	Word count 3rd quartile
Guardian & Observer	Centre-left	Quality	109	14.4	5	4.6	457	680	907
Independent & Independent on Sunday	Centre-left	Quality	61	8.1	0	-	247	474	690
Mirror & Sunday Mirror	Centre-left	Tabloid	198	26.2	2	1.0	121	219	459
Daily Telegraph & Sunday Telegraph	Centre-right	Quality	107	14.1	9	8.4	182	346	502
Daily Mail & Mail on Sunday	Centre-right	Middle-market	134	17.7	6	4.5	263	438	672
Sun	Centre-right	Tabloid	148	19.6	0	-	98	195	337
		**Total**	**757**	**100.0**	**22**	**2.9**	**151**	**325**	**595**

*percentage within whole sample
**percentage of front-page articles within publication

For peer review only - http://bmjopen.bmj.com/site/about/guidelines.xhtml

Table 2. Frequency of mentions of problem definitions, drivers, and categories of solutions

Theme	Total (n=757) n	%	Inter-rater agreement*
**Problem definitions**			
Quantifies obesity prevalence within the UK	413	54.6	0.834
Quantifies obesity prevalence elsewhere	80	10.6	0.814
Mentions increase in obesity rates	389	51.4	0.940
Mentions obesity as a risk to health	397	52.4	0.893
Mentions obesity as a cosmetic problem	23	3.0	0.850
Mentions obesity as a burden to NHS	102	13.5	0.814
Mentions obesity as an economic burden to society	32	4.2	0.630
Mentions socio-economic and geographical differences	74	9.8	0.706
Mentions women and/or girls	112	14.8	0.706
Mentions men and/or boys	56	7.4	0.706
Obesity is not a problem, over-hyped etc.	93	12.3	0.850
Mentions discrimination, bullying or stigmatisation	70	9.2	1.000
**Drivers of obesity**			
Overall drivers			
Any drivers mentioned	522	69.0	n/a**
Any biological/genetic driver mentioned	70	9.2	n/a**
Any individual driver mentioned	453	59.8	n/a**
Any societal driver mentioned	214	28.3	n/a**
Individual drivers			
Mentions poor diet, overeating	235	31.0	0.857
Mentions poor self-control, willpower or choices	60	7.9	0.680
Mentions insufficient exercise, sedentary lifestyle	224	29.6	0.919
Mentions parenting shortcomings	246	32.5	0.939
Societal drivers			
Mentions an abundance of processed/fast food	129	17.0	0.752
Mentions a lack of health services or facilities	53	7.0	0.945
Mentions food/drink advertising and promotions	90	11.9	1.000
**Solutions to obesity**			
Any solution mentioned	538	71.1	n/a**
Individual solution mentioned	276	36.5	0.920
Societal solution mentioned	214	28.3	0.839
Biological solution mentioned	52	6.9	1.000

*Cohen's kappa test of inter-rater agreement.

**Agreement was not calculated for these variables as they were computed from other, manually-coded variables

For peer review only - http://bmjopen.bmj.com/site/about/guidelines.xhtml

Table 3. Likelihood of centre-right-aligned publications mentioning definitions of obesity

	Unadjusted			Adjusted*		
	OR	95% CI	P-value	OR	95% CI	P-value
**Problem definitions**						
Quantifies obesity prevalence within the UK						
	0.97	0.73-1.30	0.858	0.92	0.67-1.27	0.608
Quantifies obesity prevalence elsewhere						
	0.63	0.40-1.01	0.057	0.59	0.34-1.03	0.065
Mentions increase in obesity rates						
	0.70	0.52-0.93	**0.014**	0.59	0.42-0.81	**0.001**
Mentions obesity as a risk to health						
	1.02	0.77-1.36	0.885	0.88	0.64-1.22	0.456
Mentions obesity as a cosmetic problem						
	0.40	0.16-0.99	**0.048**	0.35	0.11-1.05	0.061
Mentions obesity as a burden to NHS						
	0.57	0.37-0.87	**0.009**	0.50	0.30-0.83	**0.008**
Mentions obesity as an economic burden to society						
	0.36	0.19-0.70	**0.003**	0.35	0.16-0.78	**0.010**
Mentions socio-economic and geographical differences						
	0.62	0.38-1.00	0.051	0.85	0.51-1.43	0.547
Mentions women and/or girls						
	0.86	0.58-1.29	0.467	0.77	0.48-1.23	0.271
Mentions men and/or boys						
	0.59	0.34-1.03	0.062	0.43	0.22-0.88	**0.020**
Obesity is not a problem, over-hyped etc.						
	0.75	0.29-1.93	0.552	0.73	0.25-2.15	0.565
Mentions discrimination, bullying or stigmatisation						
	0.56	0.36-0.87	**0.010**	0.44	0.25-0.76	**0.003**

*Adjusted for publication market

For peer review only

Table 4. Likelihood of centre-right-aligned publications mentioning categories of driver and solution

	Unadjusted			Adjusted*		
	OR	95% CI	P-value	OR	95% CI	P-value
**Drivers of obesity**						
Overall drivers						
Any drivers mentioned	0.90	0.66-1.23	0.505	0.78	0.56-1.10	0.162
Any biological/genetic driver mentioned	0.73	0.45-1.20	0.214	0.85	0.49-1.46	0.557
Any individual driver mentioned	1.00	0.75-1.34	0.974	0.84	0.61-1.16	0.292
Any societal driver mentioned	0.62	0.45-0.86	**0.004**	0.69	0.48-0.99	**0.046**
Individual drivers						
Mentions poor diet, overeating	0.73	0.54-0.99	**0.045**	0.65	0.46-0.93	**0.018**
Mentions poor self-control, willpower or choices	0.61	0.35-1.04	0.068	0.71	0.39-1.28	0.255
Mentions insufficient exercise, sedentary lifestyle	0.75	0.55-1.03	0.077	0.67	0.47-0.97	**0.032**
Mentions parenting shortcomings	1.14	0.84-1.55	0.386	1.08	0.77-1.52	0.660
Societal drivers						
Mentions an abundance of processed/fast food	0.61	0.41-0.89	**0.011**	0.73	0.48-1.12	0.153
Mentions a lack of health services or facilities	0.90	0.52-1.58	0.725	0.87	0.46-1.65	0.671
Mentions food/drink advertising and promotions	0.56	0.36-0.88	**0.012**	0.55	0.32-0.94	**0.030**
**Solutions to obesity**						
Biological	0.73	0.42-1.29	0.286	0.54	0.26-1.09	0.087
Individual	0.90	0.67-1.20	0.464	0.90	0.64-1.25	0.527
Societal	0.62	0.46-0.83	**0.001**	0.54	0.39-0.75	**0.000**

*Adjusted for publication market

For peer review only

For peer review only

For peer review only - http://bmjopen.bmj.com/site/about/guidelines.xhtml

References

1. Ebbeling CB, Pawlak DB, Ludwig DS. Childhood obesity: public-health crisis, common sense cure. Lancet 2002;**360**(9331):473-82
2. Ng M, Fleming T, Robinson M, et al. Global, regional, and national prevalence of overweight and obesity in children and adults during 1980–2013: a systematic analysis for the Global Burden of Disease Study 2013. Lancet 2014;**384**(9945):766-81
3. NHS Digital. Statistics on Obesity, Physical Activity and Diet, 2017.
4. Scottish Government. The Scottish Health Survey: 2015 edition. Edinburgh: Scottish Government, 2015.
5. Simmonds M, Llewellyn A, Owen CG, et al. Predicting adult obesity from childhood obesity: a systematic review and meta-analysis. Obesity Reviews 2016;**17**(2):95-107 doi: 10.1111/obr.12334[published Online First: Epub Date]|.
6. HM Government. Childhood obesity: a plan for action, 2017.
7. Scottish Government. Increase the proportion of healthy weight children. Secondary Increase the proportion of healthy weight children 2016. http://www.gov.scot/About/Performance/scotPerforms/indicator/healthyweight.
8. Butland B, Jebb S, Kopelman P, et al. Foresight. Tackling Obesities: Future Choices - Project report. 2nd ed. London: Government Office for Science, 2007.
9. Friedman DA. Public health regulation and the limits of paternalism. Conn L Rev 2013;**46**:1687
10. Swinburn BA, Sacks G, Hall KD, et al. The global obesity pandemic: shaped by global drivers and local environments. Lancet 2011;**378**(9793):804-14
11. Swinburn BA. Obesity prevention: the role of policies, laws and regulations. Australia and New Zealand health policy 2008;**5**(1):12
12. Hilbert A, Rief W, Braehler E. What determines public support of obesity prevention? J Epidemiol Community Health 2007;**61**(7):585
13. Evans WD, Finkelstein EA, Kamerow DB, et al. Public perceptions of childhood obesity. Am J Prev Med 2005;**28**(1):26-32 doi: http://dx.doi.org/10.1016/j.amepre.2004.09.008[published Online First: Epub Date]|.
14. McCombs ME, Shaw DL. The agenda-setting function of mass media. Public Opin Q 1972;**36**(2):176-87
15. Scheufele DA, Tewksbury D. Framing, agenda setting, and priming: The evolution of three media effects models. J Commun 2007;**57**(1):9-20
16. Entman RM. Framing: Toward clarification of a fractured paradigm. J Commun 1993;**43**(4):51-58
17. Barry CL, Brescoll VL, Gollust SE. Framing Childhood Obesity: How Individualizing the Problem Affects Public Support for Prevention. Polit Psychol 2013:n/a-n/a doi: 10.1111/pops.12018[published Online First: Epub Date]|.
18. Gollust SE, Niederdeppe J, Barry CL. Framing the Consequences of Childhood Obesity to Increase Public Support for Obesity Prevention Policy. Am J Public Health 2013;**103**(11):e96-e102 doi: 10.2105/ajph.2013.301271[published Online First: Epub Date]|.
19. Barry CL, Brescoll VL, Brownell KD, et al. Obesity Metaphors: How Beliefs about the Causes of Obesity Affect Support for Public Policy. Milbank Q 2009;**87**(1):7-47 doi: 10.1111/j.1468-0009.2009.00546.x[published Online First: Epub Date]|.
20. Boyce T. The media and obesity. Obesity Reviews 2007;**8**:201-05 doi: 10.1111/j.1467-789X.2007.00342.x[published Online First: Epub Date]|.
21. Hilton S, Patterson C, Teyhan A. Escalating coverage of obesity in UK newspapers: the evolution and framing of the "obesity epidemic" from 1996 to 2010. Obes 2012;**20**(8):1688-95
22. Hilbert A, Ried J. Obesity in Print: An Analysis of Daily Newspapers. Obesity Facts 2009;**2**(1):46-51
23. Patterson C, Hilton S. Normalisation and Stigmatisation of Obesity in UK Newspapers: a Visual Content Analysis. The Open Obesity Journal 2013;**5**:82-91 doi: 10.2174/1876823720131001011[published Online First: Epub Date]|.

For peer review only - http://bmjopen.bmj.com/site/about/guidelines.xhtml

24. Flint SW, Hudson J, Lavallee D. The portrayal of obesity in UK national newspapers. Stigma and Health 2016;**1**(1):16
25. Islam S, Fitzgerald L. Indigenous obesity in the news: a media analysis of news representation of obesity in Australia's Indigenous population. BMC obesity 2016;**3**(1):30
26. Atanasova D. Obesity frames in British and German online newspapers, 2009-2011. Department of Media and Communication, 2015.
27. Bonfiglioli C. Overweight and Obesity in TV News. Values and Choices in Television Discourse: Springer, 2015:87-108.
28. Boero N. All the news that's fat to print: the American "obesity epidemic" and the media. Qualitative Sociology 2007;**30**(1):41-60
29. Lawrence RG. Framing obesity: The evolution of news discourse on a public health issue. Harvard International Journal of Press/Politics 2004;**9**(3):56-75 doi: citeulike-article-id:4228152[published Online First: Epub Date]|.
30. Kim S-H, Willis LA. Talking about Obesity: News Framing of Who Is Responsible for Causing and Fixing the Problem. Journal of Health Communication 2007;**12**(4):359-76 doi: 10.1080/10810730701326051[published Online First: Epub Date]|.
31. Gollust SE, Eboh I, Barry CL. Picturing obesity: Analyzing the social epidemiology of obesity conveyed through US news media images. Soc Sci Med 2012;**74**(10):1544-51
32. Jeong M, Gilmore JS, Bleakley A, et al. Local news media framing of obesity in the context of a sugar-sweetened beverage reduction media campaign. Journal of nutrition education and behavior 2014;**46**(6):583-88
33. Varava K. Visual framing, stigmatization, race, and obesity: Examining television news presentation of stories about obesity and stories about health. Communication Studies 2016;**67**(5):509-29
34. Barry CL, Jarlenski M, Grob R, et al. News media framing of childhood obesity in the United States from 2000 to 2009. Pediatrics 2011;**128**(1):132-45 doi: peds.2010-3924 [pii]

10.1542/peds.2010-3924[published Online First: Epub Date]|.
35. Hawkins KW, Linvill DL. Public health framing of news regarding childhood obesity in the United States. Health communication 2010;**25**(8):709-17
36. Bastian A. Representations of childhood obesity in Australian newsprint media and academic literature. Aust N Z J Public Health 2011;**35**(2):135-39
37. Maher J, Fraser S, Wright J. Framing the mother: childhood obesity, maternal responsibility and care. J Gend Stud 2010;**19**(3):233-47
38. Kalin SR, Fung TT. Comparison of child obesity prevention and control content in mainstream and Spanish-language US parenting magazines. Journal of the Academy of Nutrition and Dietetics 2013;**113**(1):133-40
39. Boero N. Obesity in the media: Social science weighs in. Critical Public Health 2013;**23**(3):371-80
40. Douglas F, Clark J, Craig L, et al. "It'sa balance of just getting things right": mothers' views about pre-school childhood obesity and obesity prevention in Scotland. BMC public health 2014;**14**(1):1009
41. Harris JK, Moreland-Russell S, Tabak RG, et al. Communication About Childhood Obesity on Twitter. Am J Public Health 2014;**104**(7):e62-e69 doi: 10.2105/ajph.2013.301860[published Online First: Epub Date]|.
42. Chou W-yS, Prestin A, Kunath S. Obesity in social media: a mixed methods analysis. Translational behavioral medicine 2014;**4**(3):314-23
43. Tobitt C. National newspaper ABCs: Industry-wide circulation decline continues as Metro and Sun top the table. Secondary National newspaper ABCs: Industry-wide circulation decline continues as Metro and Sun top the table 2018. https://www.pressgazette.co.uk/national-newspaper-abcs-industry-wide-circulation-decline-continues-as-metro-and-sun-top-the-table/.

For peer review only - http://bmjopen.bmj.com/site/about/guidelines.xhtml

44. Tobitt C. The Sun overtakes Mail Online to become UK's biggest online newsbrand, latest Comscore data shows. Secondary The Sun overtakes Mail Online to become UK's biggest online newsbrand, latest Comscore data shows 2018. https://www.pressgazette.co.uk/the-sun-overtakes-mail-online-to-become-uks-biggest-online-newspaper-brand-latest-comscore-data-shows/.
45. McCombs M. *Setting the agenda: Mass media and public opinion*: John Wiley & Sons, 2018.
46. Harder RA, Sevenans J, Van Aelst P. Intermedia agenda setting in the social media age: How traditional players dominate the news agenda in election times. The International Journal of Press/Politics 2017;**22**(3):275-93
47. Press Gazette. National newspaper print ABCs for Jan 2017: Times and Observer both boost print sales year on year. Secondary National newspaper print ABCs for Jan 2017: Times and Observer both boost print sales year on year 2017. http://www.pressgazette.co.uk/national-newspaper-print-abcs-for-jan-2017-observer-up-year-on-year-the-sun-is-fastest-riser-month-on-month/.
48. Ponsford D. Daily Mail and Sun launch front-page attacks on Corbyn as Fleet Street lines up behind Theresa May. Secondary Daily Mail and Sun launch front-page attacks on Corbyn as Fleet Street lines up behind Theresa May 2017. https://www.pressgazette.co.uk/daily-mail-and-sun-launch-front-page-attacks-on-corbyn-as-fleet-street-lines-up-behind-theresa-may/.
49. Smith M. How left or right-wing are the UK's newspapers? Secondary How left or right-wing are the UK's newspapers? 2017. https://yougov.co.uk/news/2017/03/07/how-left-or-right-wing-are-uks-newspapers/.
50. Kellner P. General election 2015: how Britain really voted. Secondary General election 2015: how Britain really voted 2015. https://yougov.co.uk/topics/politics/articles-reports/2015/06/08/general-election-2015-how-britain-really-voted.
51. Landis JR, Koch GG. The measurement of observer agreement for categorical data Biometrics 1977;**33**(1):159-74
52. Patterson C, Semple S, Wood K, et al. A quantitative content analysis of UK newsprint coverage of proposed legislation to prohibit smoking in private vehicles carrying children. BMC Public Health 2015;**15**:760
53. Patterson C, Katikireddi SV, Wood K, et al. Representations of minimum unit pricing for alcohol in UK newspapers: a case study of a public health policy debate. J Public Health 2014;**37**(1):40-49
54. Heuer CA, McClure KJ, Puhl RM. Obesity stigma in online news: A visual content analysis. Journal of Health Communication 2011;**16**(9):976-87
55. McClure KJ, Puhl RM, Heuer CA. Obesity in the news: Do photographic images of obese persons influence antifat attitudes? Journal of health communication 2011;**16**(4):359-71
56. Downs A. Up and down with ecology: the "issue-attention cycle". The public 1972
57. Schneider A, Ingram H. Social construction of target populations: Implications for politics and policy. Am Polit Sci Rev 1993:334-47
58. Freeman B, Chapman S, Storey P. Banning smoking in cars carrying children: an analytical history of a public health advocacy campaign. Aust N Z J Public Health 2008;**32**(1):60-65
59. Hilton S, Wood K, Bain J, et al. Newsprint coverage of smoking in cars carrying children: a case study of public and scientific opinion driving the policy debate. BMC Public Health 2014;**14**(1):1116
60. Schwartz MB, Puhl R. Childhood obesity: a societal problem to solve. Obesity reviews 2003;**4**(1):57-71
61. Buckton CH, Patterson C, Hyseni L, et al. The palatability of sugar-sweetened beverage taxation: A content analysis of newspaper coverage of the UK sugar debate. PLOS ONE 2018;**13**(12):e0207576 doi: 10.1371/journal.pone.0207576[published Online First: Epub Date]|.

For peer review only - http://bmjopen.bmj.com/site/about/guidelines.xhtml

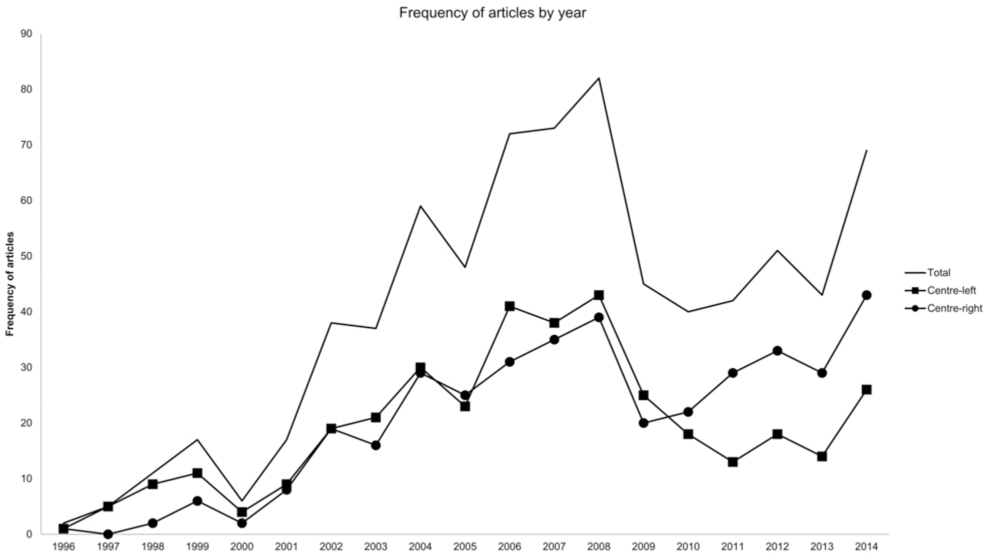

Figure 1. Frequency of articles by year

84x47mm (300 x 300 DPI)

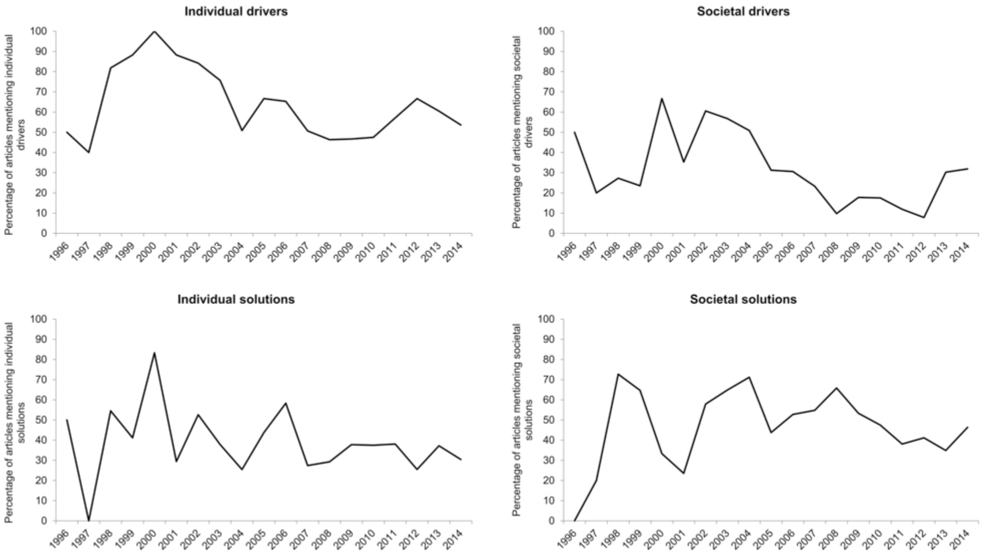

Figure 2. Trends in individual and societal drivers and solutions

84x47mm (300 x 300 DPI)

Supplementary figure 1 – Caption <Frequency of newspaper articles about generic, childhood, and adult obesity >

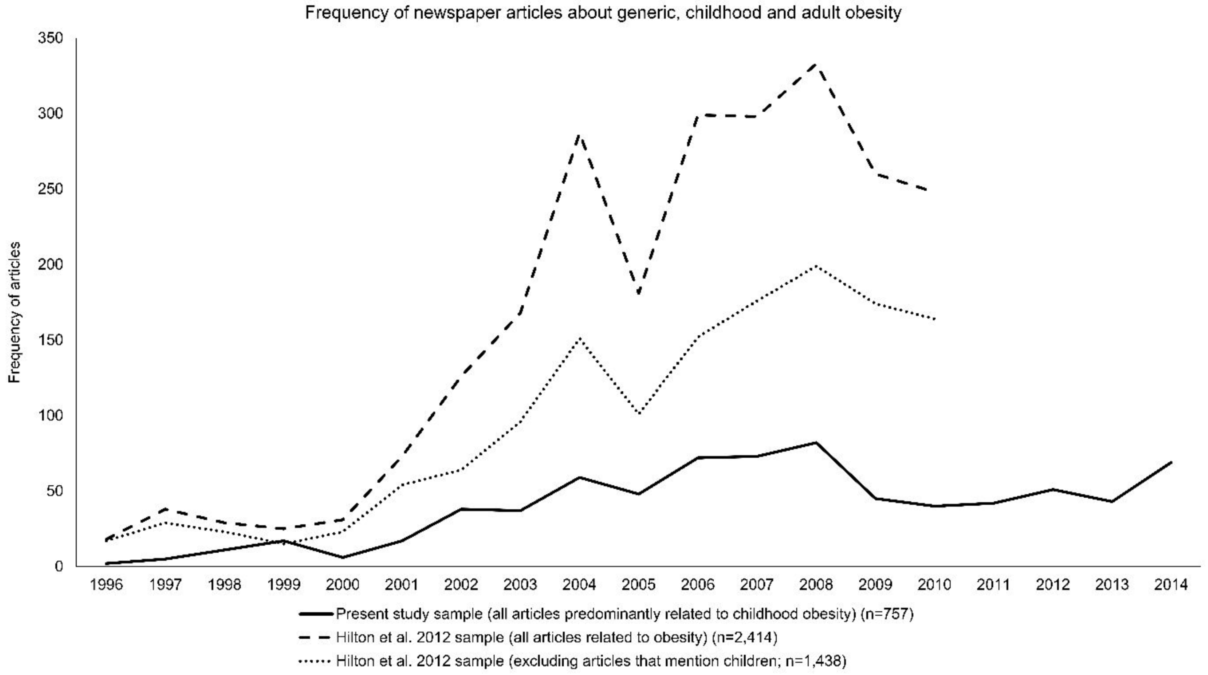

Supplementary figure 2 – Caption < Proportion of articles within years mentioning different categories of drivers and solutions about generic, childhoood, and adult obesity>

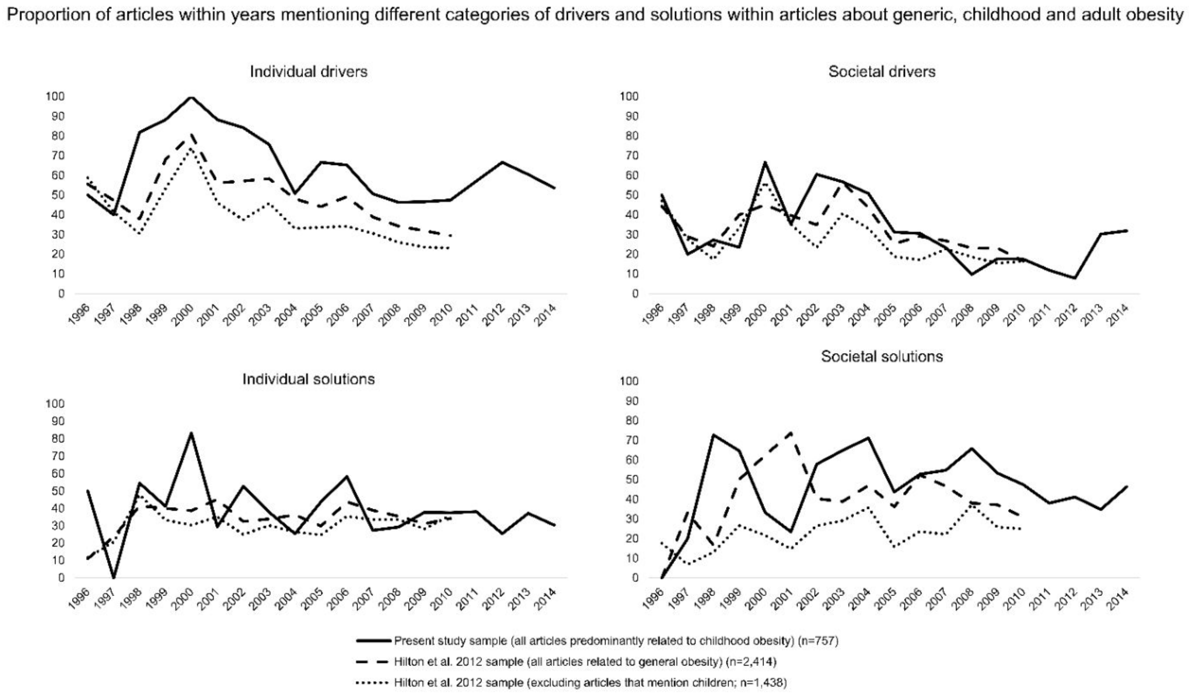

No	Item	Guide and description	Reported on page
**1**	Aim	State the research question the synthesis addresses.	Title and 8
**2**	Synthesis methodology	Identify the synthesis methodology or theoretical framework which underpins the synthesis, and describe the rationale for choice of methodology *(e.g. meta-ethnography, thematic synthesis, critical interpretive synthesis, grounded theory synthesis, realist synthesis, meta-aggregation, meta-study, framework synthesis).*	Title and 8
**3**	Approach to searching	Indicate whether the search was pre-planned (*comprehensive search strategies to seek all available studies)* or iterative (*to seek all available concepts until they theoretical saturation is achieved)*.	8,9,10
**4**	Inclusion criteria	Specify the inclusion/exclusion criteria *(e.g. in terms of population, language, year limits, type of publication, study type).*	9
**5**	Data sources	Describe the information sources used (e.g. *electronic databases (MEDLINE, EMBASE, CINAHL, psycINFO, Econlit), grey literature databases (digital thesis, policy reports), relevant organisational websites, experts, information specialists, generic web searches (Google Scholar) hand searching, reference lists)* and when the searches conducted; provide the rationale for using the data sources.	9

No	Item	Guide and description	Reported on page
**6**	Electronic Search strategy	Describe the literature search *(e.g. provide electronic search strategies with population terms, clinical or health topic terms, experiential or social phenomena related terms, filters for qualitative research, and search limits).*	9
**7**	Study screening methods	Describe the process of study screening and sifting *(e.g. title, abstract and full text review, number of independent reviewers who screened studies).*	9
**8**	Study characteristics	Present the characteristics of the included studies *(e.g. year of publication, country, population, number of participants, data collection, methodology, analysis, research questions).*	9
**9**	Study selection results	Identify the number of studies screened and provide reasons for study exclusion *(e,g, for comprehensive searching, provide numbers of studies screened and reasons for exclusion indicated in a figure/flowchart; for iterative searching describe reasons for study exclusion and inclusion based on modifications t the research question and/or contribution to theory development).*	9
**10**	Rationale for appraisal	Describe the rationale and approach used to appraise the included studies or selected findings *(e.g. assessment of conduct (validity and robustness), assessment of reporting*	10

No	Item	Guide and description	Reported on page
		(transparency), assessment of content and utility of the findings).	
**11**	Appraisal items	State the tools, frameworks and criteria used to appraise the studies or selected findings *(e.g. Existing tools: CASP, QARI, COREQ, Mays and Pope* [25]*; reviewer developed tools; describe the domains assessed: research team, study design, data analysis and interpretations, reporting).*	10
**12**	Appraisal process	Indicate whether the appraisal was conducted independently by more than one reviewer and if consensus was required.	10
**13**	Appraisal results	Present results of the quality assessment and indicate which articles, if any, were weighted/excluded based on the assessment and give the rationale.	NA
**14**	Data extraction	Indicate which sections of the primary studies were analysed and how were the data extracted from the primary studies? *(e.g. all text under the headings "results /conclusions" were extracted electronically and entered into a computer software).*	10
**15**	Software	State the computer software used, if any.	10
**16**	Number of reviewers	Identify who was involved in coding and analysis.	10

No	Item	Guide and description	Reported on page
**17**	Coding	Describe the process for coding of data *(e.g. line by line coding to search for concepts).*	10
**18**	Study comparison	Describe how were comparisons made within and across studies *(e.g. subsequent studies were coded into pre-existing concepts, and new concepts were created when deemed necessary).*	11
**19**	Derivation of themes	Explain whether the process of deriving the themes or constructs was inductive or deductive.	NA
**20**	Quotations	Provide quotations from the primary studies to illustrate themes/constructs, and identify whether the quotations were participant quotations of the author's interpretation.	NA
**21**	Synthesis output	Present rich, compelling and useful results that go beyond a summary of the primary studies (e.g. *new interpretation, models of evidence, conceptual models, analytical framework, development of a new theory or construct).*	15-19?
